# Small Extracellular Vesicles Derived from Altered Peptide Ligand‐Loaded Dendritic Cell Act as A Therapeutic Vaccine for Spinal Cord Injury Through Eliciting CD4^+^ T cell‐Mediated Neuroprotective Immunity

**DOI:** 10.1002/advs.202304648

**Published:** 2023-11-30

**Authors:** Sikai Wang, Guanglei Li, Xiongjie Liang, Zexuan Wu, Chao Chen, Fawang Zhang, Jiawen Niu, Xuefeng Li, Jinglong Yan, Nanxiang Wang, Jing Li, Yufu Wang

**Affiliations:** ^1^ Department of Orthopedic Surgery Second Affiliated Hospital of Harbin Medical University No. 246 Baojian Road Harbin 150086 China; ^2^ Heilongjiang Provincial Key Laboratory of Hard Tissue Development and Regeneration The Second Affiliated Hospital of Harbin Medical University No. 246 Baojian Road Harbin 150086 China; ^3^ Faculty of Medicine and Dentistry University of Alberta Edmonton T5C 0T2 Canada; ^4^ Department of Pathology and Electron Microscopy Faculty of Basic Medical Science Harbin Medical University No. 157 Baojian Road Harbin 150086 China

**Keywords:** antigen‐specific immune, dendritic cells, neuroprotective immunity, small extracellular vesicles, spinal cord injury

## Abstract

The balance among different CD4^+^ T cell subsets is crucial for repairing the injured spinal cord. Dendritic cell (DC)‐derived small extracellular vesicles (DsEVs) effectively activate T‐cell immunity. Altered peptide ligands (APLs), derived from myelin basic protein (MBP), have been shown to affect CD4^+^ T cell subsets and reduce neuroinflammation levels. However, the application of APLs is challenging because of their poor stability and associated side effects. Herein, it is demonstrate that DsEVs can act as carriers for APL MBP_87‐99_A^91^ (A91‐DsEVs) to induce the activation of 2 helper T (Th2) and regulatory T (Treg) cells for spinal cord injury (SCI) in mice. These stimulated CD4^+^ T cells can efficiently “home” to the lesion area and establish a beneficial microenvironment through inducing the activation of M2 macrophages/microglia, inhibiting the expression of inflammatory cytokines, and increasing the release of neurotrophic factors. The microenvironment mediated by A91‐DsEVs may enhance axon regrowth, protect neurons, and promote remyelination, which may support the recovery of motor function in the SCI model mice. In conclusion, using A91‐DsEVs as a therapeutic vaccine may help induce neuroprotective immunity in the treatment of SCI.

## Graphical abstract

This study uses Dendritic cell‐derived small extracellular vesicles (DsEVs) loaded with APL MBP_87‐99_A^91^ (A91‐DsEVs). A91‐DsEVs promotes activation of Th2 and Treg cells, which can home to the injured spinal cord and induce neuroprotective immunity. The treatment with A91‐DsEVs can enhance axon regrowth, neuronal survival, and remyelination, which may support the functional motor recovery in the SCI model mice.

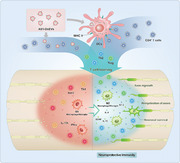

## 1 Introduction

Spinal cord injury (SCI) is a severe form of central nervous system (CNS) damage that leads to significant neurological dysfunction due to disruption of neural circuitry.^[^ ^1^ ^]^ The post‐SCI pathophysiological progression involves an immune response.^[^ ^2^ ^]^ CD4^+^ T cells are important components of the immune response following an injury, and the balance between different subtypes of CD4^+^ T cells is crucial for neuroprotection.^[^ ^3^ ^]^

During the immune response after SCI, most CD4^+^ T cells are biased towards T helper type 1 (Th1) and T helper type 17 (Th17) cells. They induce an increased release of inflammatory cytokines and autoantibodies. This process persists for days or weeks, and aggravates neuronal loss, demyelination and axonal damage.^[^ ^4^, ^5^, ^6^ ^]^ In contrast, 2 helper T (Th2) and regulatory T (Treg) cells have been shown to inhibit the over‐activation of inflammatory response.^[^ ^7^ ^]^ Studies have highlighted the significant role of Th2 cells in providing protection and promoting recovery after SCI by secreting anti‐inflammatory cytokines.^[^ ^8^, ^9^, ^10^ ^]^ Treg cells have been shown to play an anti‐inflammatory role and to contribute to remyelination and axonal survival after SCI.^[^ ^11^, ^12^ ^]^ Therefore, CD4^+^ T‐cell regulation is an important target for SCI treatment.^[^ ^7^ ^]^

A promising strategy for changing T‐cell subsets is the use of altered peptide ligands (APLs) derived from myelin sheath antigens with mutations in critical T‐cell receptor (TCR) or major histocompatibility complexes (MHC) contact residues. APLs are analogs derived from native peptides that commonly carry amino acid substitutions at TCR contact residues.^[^ ^13^ ^]^ One such antigen is APL of myelin basic protein (MBP).^[^ ^14^ ^]^ A specific segment of MBP consisting of amino acids 87–99 (MBP_87–99_) has been shown to be a major target of T cells in neuroinflammation. Previous studies have demonstrated that MBP_87‐99_A^91^, an APL of MBP_87‐99_ in which the lysine residue at position 91 is replaced with alanine, can alter the polarity of inflammatory CD4^+^ T cells and induce neuroprotective effect.^[^ ^15^, ^16^ ^]^ However, the clinical application of APLs, such as MBP_87‐99_A^91^, comes with many challenges, including poor stability due to enzymatic degradation in blood circulation^[^ ^17^ ^]^ and damaging biological barriers that result in toxicity to tissues and organs.^[^ ^18^ ^]^ APLs may also cause hypersensitivity responses because of their commonly used high concentrations.^[^ ^19^ ^]^

Dendritic cell (DC)‐derived small extracellular vesicles (DsEVs) are ideal carriers for peptides to induce antigen‐specific immunity, the stable lipid bilayers effectively protect their contents and reduce antigen cargo loss.^[^ ^20^, ^21^ ^]^ DsEVs contain MHC I and MHC II, along with costimulatory molecules that facilitate peptide presentation and T‐cell activation.^[^ ^22^ ^]^ DsEVs have been shown to be non‐immunogenic or possess very low immunogenicity.^[^ ^23^ ^]^ The therapeutic potential of antigen‐specific immunity induced by DsEVs has mainly been evaluated in treating conditions such as tumors and infectious diseases.^[^ ^24^, ^25^, ^26^, ^27^ ^]^

**Scheme 1:**
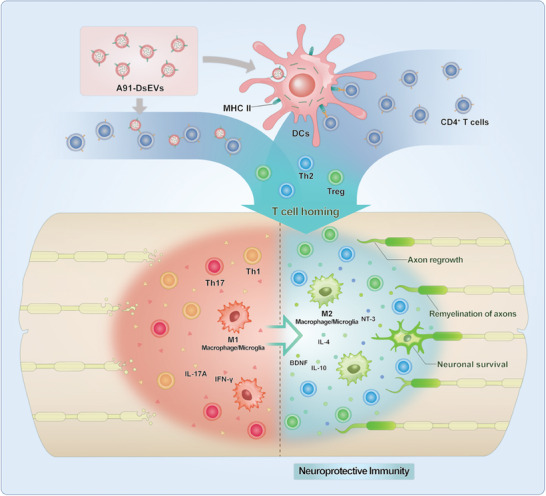
Small extracellular vesicles derived from dendritic cells (DsEVs) loaded with MBP_87‐99_A^91^ (A91‐DsEVs) was used for spinal cord injury treatment. A91‐DsEVs promoted activation of Th2 and Treg cells, which can home to the injured spinal cord and induce neuroprotective immunity. The treatment with A91‐DsEVs can enhance axon regrowth, neuronal survival and remyelination, which may support the functional motor recovery in the SCI model mice.

However, the application of DsEVs for the treatment of CNS injuries remains underexplored. For SCI treatment, DsEVs as a therapeutic vaccine may offer several advantages: 1) Compared with other nanomaterials (liposomes or nanoparticles, among others), DsEVs can deliver the peptide cargo directly to the lymphoid organs, antigen‐presenting cells or T cells, boosting immunotherapeutic efficacy;^[^ ^28^ ^]^ 2) DsEVs can stimulate antigen‐specific T‐cell activation, mitigating the risk of side effects from the generalized immune response;^[^ ^29^, ^30^ ^]^ 3) DsEVs could deliver their therapeutic benefits through peripheral injection, avoiding secondary damage to the lesion area caused by local delivery. Hence, we aimed to design a DsEVs‐based nanovaccine platform to induce a CD4^+^ T‐cell‐mediated neuroprotective effect at the injury site and to demonstrate the potential of this innovative approach for SCI treatment (**Scheme** **1**).

## 2 Results

### 2.1 Characterization of A91‐DsEVs

**Figure 1:**
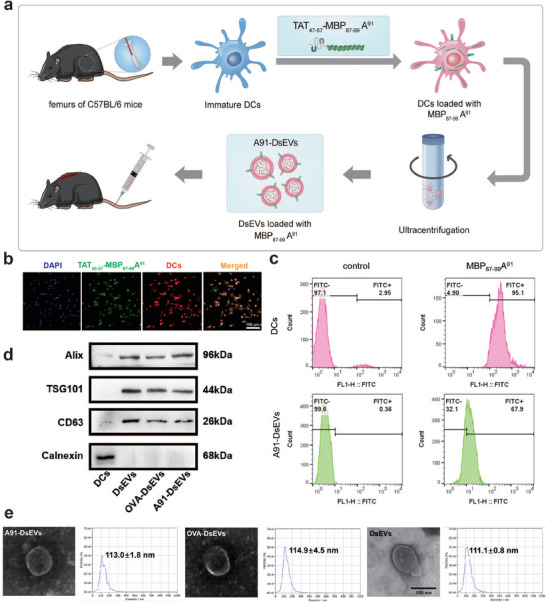
Successful isolation of A91‐DsEVs from DCs culture medium. a) Schematic illustration of A91‐DsEVs isolation. b) Immunofluorescence images to show FITC‐labeled TAT_47‐57_‐MBP_87‐99_A^91^ (green) loaded into DCs (red) (Scale bar = 100 µm, *n* = 3). c) Flow cytometry to quantify the loading rate of MBP_87‐99_A^91^ in DCs (pink) and A91‐DsEVs (green) (*n* = 3). d) Western blotting to detect DsEVs positive markers Alix, TSG101, CD63 and negative marker Calnexin in DCs and A91‐DsEVs (*n* = 3). e) TEM to visualize ultrastructure of DsEVs (Scale bar = 100 nm). NTA to reveal the size distribution of DsEVs (*n* = 3). Data were expressed as mean ± standard deviation (SD).

In this study, we introduced MBP_87‐99_A^91^ or ovalbumin (OVA) as vaccine cargo into DsEVs. TAT_47‐57_, a cell‐penetrating peptide (CPP), was added to peptide to enhance the loading efficiency. **Figure** **1**a illustrates the procedure used for A91‐DsEVs isolation. Immunofluorescence staining showed clear internalization of isothiocyanate (FITC)‐labeled TAT_47‐57_‐MBP_87‐99_A^91^ in DCs (Figure 1b). The loading efficiency of MBP_87‐99_A^91^ peptide into DCs and DsEVs was quantified using flow cytometry. The result showed that MBP_87‐99_A^91^ peptide (labeled with FITC) was successfully loaded into DCs and A91‐DsEVs at loading rates of 95.1% and 67.9% respectively (Figure 1c). Flow cytometry was used to assess the expression of MHC II and costimulatory molecules in DCs after loading with MBP_87‐99_A^91^ (Figure S1, Supporting Information). The results revealed the expression of MHC II, CD86 and CD80 were raised in MBP_87‐99_A^91^‐treated group and OVA‐treated group compared to that in the PBS‐treated group.

Western blotting was used to investigate the characteristic membrane protein markers of A91‐DsEVs, including the positive markers, Alix, TSG101, and CD63, and the negative marker Calnexin (Figure 1d). Alix, TSG101 and CD63 expression was detected in A91‐DsEVs, OVA‐DsEVs, and DsEVs. Calnexin was only detected in DCs. Next, vesicle morphology of different groups of DsEVs was characterized through transmission electron microscopy (TEM) (Figure 1e). TEM revealed vesicles with typical spherical vesicular structures. To investigate the size distribution profile of DsEVs, we performed a size detection using nanoparticle tracking analysis (NTA) (Figure 1e). NTA revealed the particles of A91‐DsEVs have a peak diameter of 113.0±1.8 nm, which was similar to that of other groups (OVA‐DsEVs: 114.9±4.5 nm; DsEVs: 111.1±0.8 nm.). These findings indicated the successful isolation of A91‐DsEVs from DCs culture medium.

### 2.2 A91‐DsEVs Could be Internalized by DCs and CD4^+^ T Cells and Induce the Polarization of CD4^+^ T Cells toward Th2 and Treg Subtypes In Vitro

**Figure 2:**
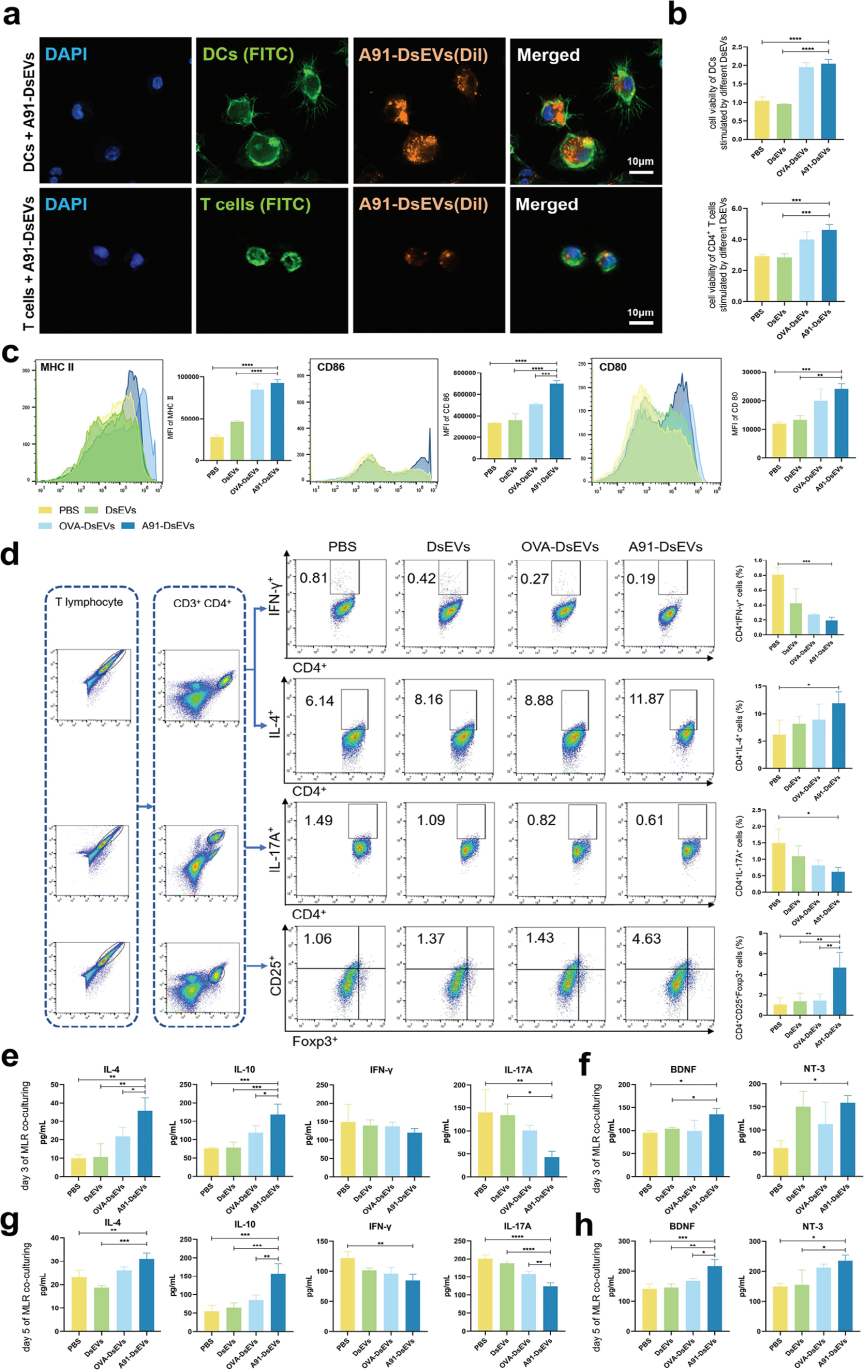
A91‐DsEVs internalized by DCs and CD4^+^ T cells, increased the antigen‐presenting capability of DCs and regulated CD4^+^ T cells polarization toward anti‐inflammatory phenotype. a) Images to depict A91‐DsEVs (orange) internalized by DCs (green) and CD4^+^ T cells (green). b) The CCK‐8 assay to assess the cell viability of DCs and CD4^+^ T cells derived from distinct treatment groups, which involved co‐cultivation of different DsEVs or PBS with DCs and CD4^+^ T cells respectively (*n* = 3). c) Flow cytometry of MHC II, CD86, and CD80 expression in DCs following treatment with A91‐DsEVs, OVA‐DsEVs, DsEVs, or PBS (*n* = 3). d) Flow cytometry to show the impact of A91‐DsEVs on the polarization of CD4^+^ T cells on day 3 of co‐culture (*n* = 3). e) ELISA to measure IL‐4, IL‐10, IFN‐γ, and IL‐17A levels in the supernatant of the MLR on day 3 of co‐culturing (*n* = 3). f) ELISA to measure NT‐3 and BDNF expression in the MLR system supernatant among different groups on day 3 (*n* = 3). g) and h) ELISA to measure IL‐4, IL‐10, IFN‐γ, and IL‐17A levels g), nerve growth factors levels (BDNF and NT‐3) h), in the supernatant of the MLR on day 5 of co‐culturing (*n* = 3). MFI, mean fluorescence intensity. Data were expressed as mean ± SD. (*p < 0.05, **p < 0.01, ***p < 0.001, ****p < 0.0001 as assessed by one‐way ANOVA with Dunnett's multiple comparisons).

To evaluate the A91‐DsEVs internalization by DCs and CD4^+^ T cells, we labeled A91‐DsEVs with 1,1‐dioctadecyl‐3,3,3,3‐tetramethylindocarbocyanine iodide (Dil). We then co‐cultured Dil‐labeled A91‐DsEVs with DCs and CD4^+^ T cells respectively. After 24 h of co‐culture, DCs were collected for further experiments. As shown in **Figure** **2**a, A91‐DsEVs (indicated by orange fluorescence) were internalized by DCs and CD4^+^ T cells. The viability of A91‐DsEVs‐treated and OVA‐DsEVs‐treated DCs was much higher than those of the DsEVs‐treated and PBS‐treated groups (Figure 2b). Similarly, the viability of CD4^+^ T cells was higher in the A91‐DsEVs‐treated and OVA‐DsEVs‐treated groups than in the PBS‐treated group (Figure 2b). Flow cytometry was used to assess the expression of MHC II and costimulatory molecules in the DCs (Figure 2c). The expression of MHC II and CD80 in DCs was higher in the A91‐DsEVs‐treated group than in the DsEVs‐ and PBS‐treated groups. The results also revealed that CD86 expression raised significantly in A91‐DsEVs‐treated group than that in the other groups. These findings demonstrate that A91‐DsEVs were efficiently internalized by DCs and CD4^+^ T cells, resulting in the upregulated expression of MHC II and costimulatory molecules on DCs and enhanced cellular viability.

We conducted quantitative real‐time PCR (qRT‐PCR) analysis of DCs co‐cultured with different DsEVs or PBS for 24 h to investigate how A91‐DsEVs regulate MHC‐II expression. The key molecules regulating MHC‐II expression were assessed, including MHC class II transactivator (CIITA), regulatory factor X 5 (RFX5), RFX‐associated protein (RFXAP) and RFX‐associated ankyrin‐containing protein (RFXANK). The results showed a significant increase in CIITA gene expression in DCs treated with A91‐DsEVs (Figure S2a, Supporting Information). This was further confirmed by an Enzyme‐linked immunosorbent assay (ELISA), which demonstrated a substantial increase in CIITA protein levels following A91‐DsEVs treatment (Figure S2b, Supporting Information).

Moreover, to simulate the immune response in vivo, we established an in vitro mixed lymphocyte reaction (MLR) system by co‐culturing DsEVs, DCs and splenic T cells. We first assed the cell viability of CD4^+^ T cells on day 3 in the MLR using the Cell Counting Kit‐8 (CCK‐8) assay. The result demonstrated that the viability of CD4^+^ T cells in A91‐DsEVs‐treated group was much higher than that in the other groups (Figure S3a, Supporting Information). Flow cytometry was used to detect cell subtype changes in splenic CD4^+^ T cells in the MLR on day 3 of co‐culturing. The results revealed decreased counts of IFN‐γ^+^ CD4^+^ and IL‐17A^+^ CD4^+^ T cells, but increased counts of Foxp3^+^ CD4^+^ and IL‐4^+^ CD4^+^ T cells in the A91‐DsEVs‐treated group (Figure 2d). Furthermore, on day 3 of co‐culture, the supernatant of the MLR system was collected to assess the levels of signature cytokines in different subsets of CD4^+^ T cells. The A91‐DsEVs group showed elevated levels of cytokines IL‐4 and IL‐10, along with decreased expression of cytokines IL‐17A and IFN‐γ (Figure 2e). The isotype used for flow cytometry is shown in Figure S4 (Supporting Information). Overall, these results underscore the potential of A91‐DsEVs to increase the antigen‐presenting capability of DCs and to regulate the polarization of CD4^+^ T cells towards Th2 and Treg subtypes in vitro.

Because CD4^+^ T cells are also a major source of nerve growth factors, including brain‐derived neurotrophic factor (BDNF) and neurotrophins‐3 (NT‐3),^[^ ^31^ ^]^ we assessed the levels of BDNF and NT‐3 in MLR using ELISA. The levels of BDNF and NT‐3 were higher in the A91‐DsEVs‐treated group than in the PBS‐treated group (Figure 2f).

To evaluate the immunological response, we also assessed T‐cell proliferation and cytokine production rates in the experimental groups on day 5 post‐MLR. The CCK‐8 assay revealed enhanced viability of CD4^+^ T cells in the A91‐DsEVs‐treated group compared to those in the other three groups (Figure S3b, Supporting Information). In addition, the A91‐DsEVs group exhibited elevated levels of IL‐4 and IL‐10, accompanied by a reduced expression of IL‐17A and IFN‐γ cytokines, compared to those observed in the other groups (Figure 2g). The levels of BDNF and NT‐3 in the MLR were higher in the A91‐DsEVs group than in the other groups on day 5 after co‐culturing (Figure 2h).

### 2.3 A91‐DsEVs Regulated the Polarization of CD4^+^ T Cells In Vivo and Promoted CD4^+^ T Cells Infiltrating to the Injury Site

**Figure 3:**
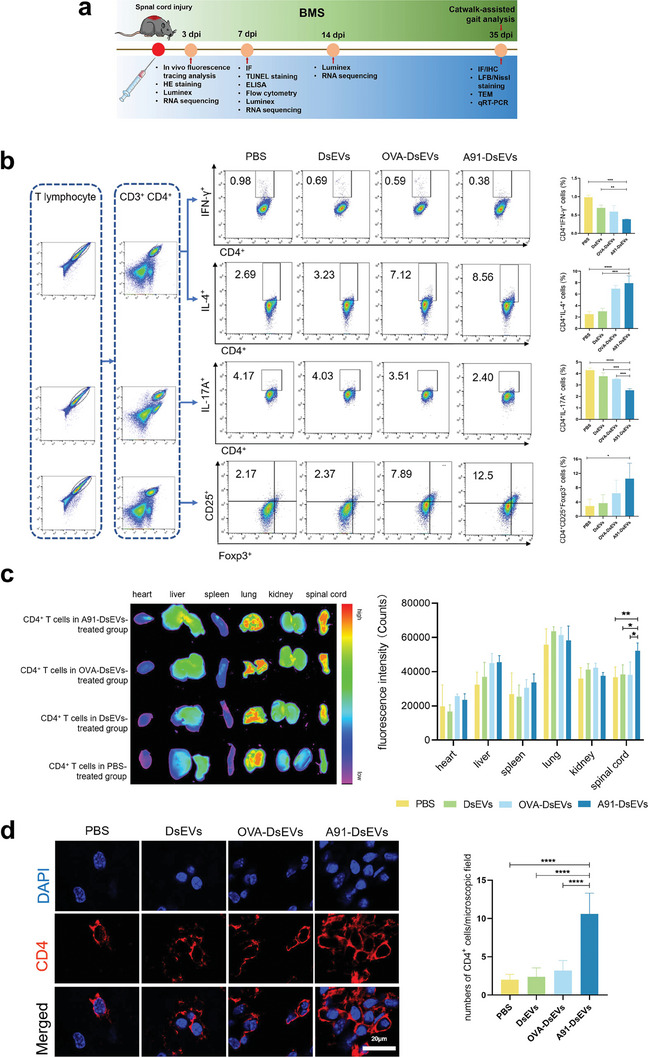
A91‐DsEVs regulated the polarization of CD4^+^ T cells in vivo and facilitated their infiltration into the injury site. a) Schematic representation of the in vivo experimental design. b) Flow cytometry of splenic CD4^+^ T cells from mice in different groups to show the modulation ability of A91‐DsEVs on CD4^+^ T cell polarization at 3 dpi (*n* = 3). c) Fluorescence tracing analysis of Dil‐labeled CD4^+^ T cells from mice in different groups in major organs 24 h post‐injection (*n* = 3). d) Immunofluorescence images to show the distribution of CD4^+^ T cells at 7 dpi. The numbers of CD4^+^ T cells were quantified from 5 microscopic fields around the injured site for each sample. (Scale Bar = 20 µm, *n* = 5). Data were expressed as mean ± SD. (*p < 0.05, **p < 0.01, ***p < 0.001, ****p < 0.0001 as assessed by One‐way ANOVA with Dunnett's multiple comparisons).

**Figure** **3**a shows a schematic of the in vivo experimental procedure. We initially labeled DsEVs from various groups with dimethylindole Red (DIR) and administered them to the mice via the tail vein, using PBS as a control. After a 6‐h interval post‐injection, we collected the spinal cord, heart, liver, spleen, lungs, and kidneys for fluorescence tracking analysis. Notably, no detectable signal was observed in the spinal cord or other major organs, whereas fluorescence was primarily localized in the spleen and liver (Figure S5, Supporting Information). This pattern of DsEVs accumulation in the liver is consistent with that previously reported.^[^ ^32^ ^]^

To further analyze the effect of A91‐DsEVs on the polarization of splenic CD4^+^ T cells in vivo, we first isolated splenic T cells from mice in different groups at 3 dpi and determined the subtype of splenic CD4^+^ T cells using flow cytometry. The A91‐DsEVs‐treated group revealed increased IL‐4^+^CD4^+^ and CD25^+^Foxp3^+^CD4^+^ T‐cell counts and a reduced IFN‐γ^+^CD4^+^ and IL‐17A^+^CD4^+^ T‐cell counts (Figure 3b). The isotype used for flow cytometry is shown in Figure S4 (Supporting Information). This result confirmed that immunization with A91‐DsEVs in SCI mice could regulate the polarization of CD4^+^ T cells towards Th2 and Treg ant‐inflammatory phenotypes.

To demonstrate the recruitment ability of splenic CD4^+^ T cells stimulated by A91‐DsEVs toward the injury site, we extracted splenic CD4^+^ T cells from mice following treatment with different groups of DsEVs or PBS. These cells were labeled with Dil dye and subsequently introduced into SCI mice via tail vein injection. Fluorescence tracing analysis performed 24 h post‐injection showed a higher intensity of Dil fluorescence within the spinal cord of A91‐DsEVs‐treated mice compared to those observed in other groups (Figure 3c). The intensity of Dil fluorescence was much higher in the spinal cord and lungs than in other organs. Immunofluorescence of spinal cord at 7 dpi was performed and the result revealed that more CD4^+^ T cells were recruited to the lesion area in the A91‐DsEVs‐treated group than in other groups (Figure 3d). These results indicate that CD4^+^ T cells stimulated by A91‐DsEVs may migrate to the injured site of spinal cord.

Local spinal cord antigen exposure is the main driver behind the recruitment of antigen‐specific CD4^+^ T cells to the injury site.^[^ ^33^ ^]^ We investigated the homing mechanism by examining the expression of activation markers (CD25, CD69) and chemokine receptors (CCR3, CCR4, CCR5, CCR6). The highest expression levels of CD25 and CD69 were observed in the A91‐DsEVs group (Figure S6a, Supporting Information). Within the A91‐DsEVs group, CCR4 gene expression was significantly increased. However, no significant differences were observed in the expression levels of CCR3, CCR5, or CCR6, among the four groups (Figure S6b, Supporting Information).

### 2.4 A91‐DsEVs Led to the Infiltration of Polarized CD4^+^ T Cells and the Polarization of Macrophages/Microglia in the Lesion Site

**Figure 4:**
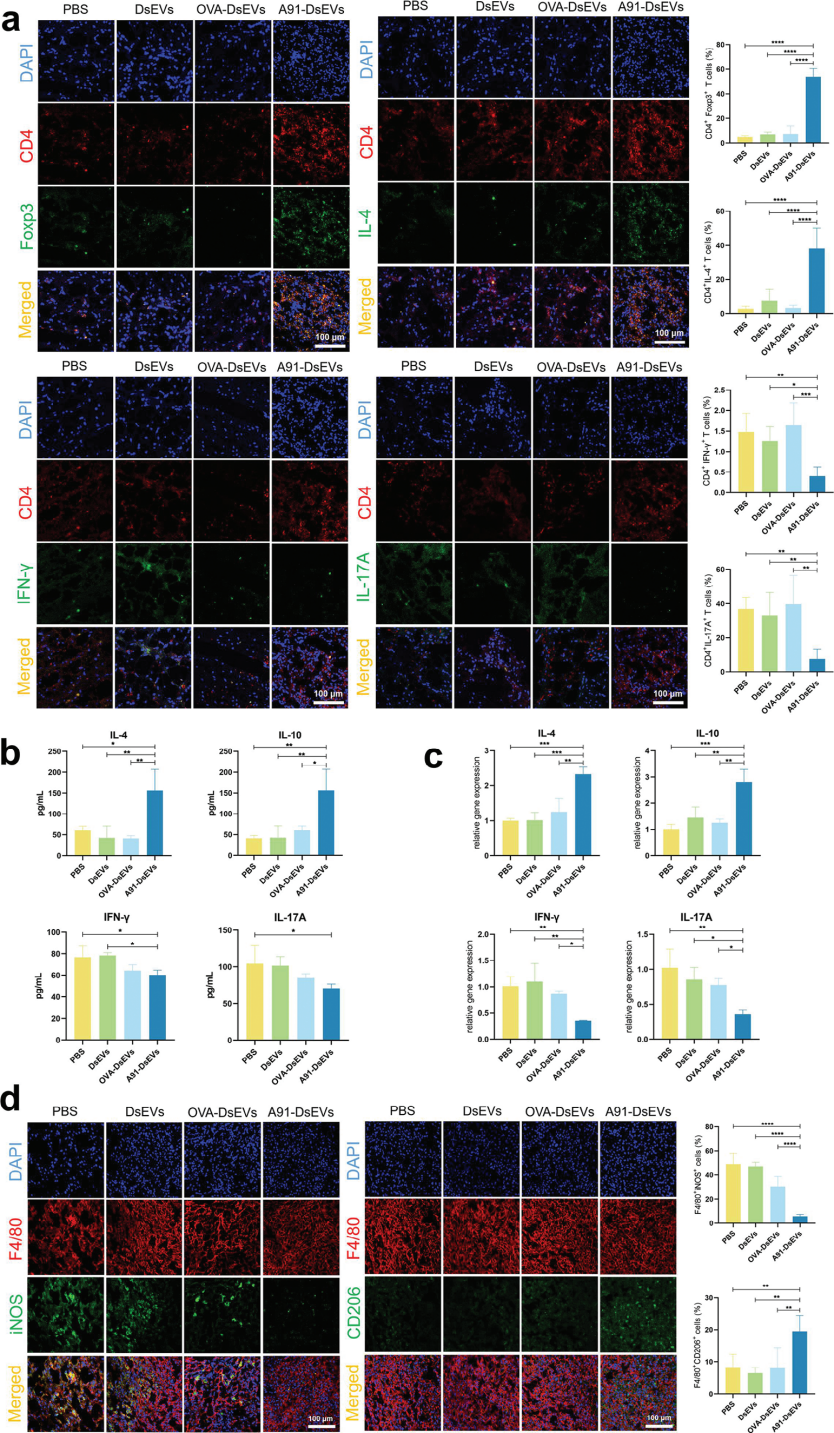
A91‐DsEVs led to the infiltration of polarized CD4^+^ T cells and the polarization of macrophages/microglia in lesion site. a) Immunofluorescence images to show the distribution of distinct subsets of CD4^+^ T cells in the injury site at 7 dpi. The bar chart depicts the proportions of different subtype cells to CD4^+^ T cells in different groups. (Scale Bar = 100 µm, *n* = 5) b) ELISA to measure the expression levels of major inflammatory cytokines in injured spinal cord from different groups at 7 dpi. c) qRT‐PCR results to show the mRNA expression level of IL‐4 and IL‐10, IFN‐γ and IL‐17A in the injured spinal cord at 7 dpi. d) Immunofluorescence images to show the distribution of different subtypes of macrophages/microglia in the injury site at 7 dpi. The bar chart showed the ratio of iNOS^+^F4/80^+^ cells and CD206^+^F4/80^+^ cells to F4/80^+^ cells in different groups. (Scale Bar = 100 µm, *n* = 5). Data were expressed as mean ± SD. (*p < 0.05, **p < 0.01, ***p < 0.001, ****p < 0.0001 as assessed by One‐way ANOVA with Dunnett's multiple comparisons).

To analyze the local immune microenvironment established by CD4^+^ T cells, the aggregation of distinct CD4^+^ T cells in the spinal cord was evaluated at 7 dpi using immunofluorescence (**Figure** **4**a). Remarkably, the A91‐DsEVs‐treated group exhibited significantly increased aggregation of CD25^+^Foxp3^+^ CD4^+^ T cells and IL‐4^+^ CD4^+^ T cells compared to those seen in the other groups. Conversely, in the A91‐DsEVs treated group, the accumulation of IFN‐γ^+^ CD4^+^ T cells was diminished and that of IL‐17A^+^ CD4^+^ T cells was reduced.

Subsequently, ELISA was utilized to measure the expression levels of IL‐4, IL‐10, IFN‐γ, IL‐17A at the injury area at 7 dpi (Figure 4b). IL‐4 and IL‐10 levels were higher in the A91‐DsEVs‐treated group than in the other groups. Conversely, the levels of IL‐17A and IFN‐γ were lower in the A91‐DsEVs‐treated group. Furthermore, the relative gene expression analysis in the injured spinal cord at 7 dpi revealed an upregulation of IL‐4 and IL‐10 expression in the A91‐DsEVs‐treated group with concurrent downregulation of IFN‐γ and IL‐17A expression (Figure 4c). Collectively, these findings indicate that A91‐DsEVs may contribute to the infiltration of Th2 and Treg cells at the lesion site.

Macrophages/microglia are important responders to SCI and peak at 7 dpi. The polarization state of macrophages/microglia could be regulated by Th2 or Treg cells.^[^ ^34^ ^]^ Therefore, their phenotype were investigated by assessing the expression of iNOS, a marker associated with M1‐type macrophages/microglia. In addition, the expression of CD206, a marker of M2‐type macrophages/microglia, was evaluated (Figure 4d). Following A91‐DsEVs treatment, a significant decrease in the population of iNOS^+^ macrophages/microglia was observed, compared to that observed with other treatments. Conversely, the population of CD206^+^ macrophages/microglia exhibited a notable increase in the A91‐DsEVs‐treated group relative to that in the other groups. To further elucidate the phenotype of the microglial recruited to the injury site, we detected the microglial marker Iba‐1 using immunofluorescence. The results demonstrated a marked reduction in the population of M1‐type microglia in the A91‐DsEVs treatment group, in contrast to the finding in the other three groups. Concurrently, there was a notable increase in the proportion of M2‐type microglia in A91‐DsEVs‐treated group (Figure S7a and b, Supporting Information).

Furthermore, we performed qRT‐PCR analysis to assess the mRNA expression levels of additional markers associated with M1‐type (IL‐1β and IL‐6) and M2‐type (Arg‐1 and CD206) microglia/macrophages. The results revealed that the A91‐DsEVs treatment group exhibited decreased expression of genes related to M1‐type markers, compared to that in the other three groups, while displaying elevated expression of genes related to M2‐type markers (Figure S7c, Supporting Information), suggesting that A91‐DsEVs induced dominant activation of M2 macrophages/microglia.

### 2.5 A91‐DsEVs Suppressed the Inflammatory Signals at the Injury Site

**Figure 5:**
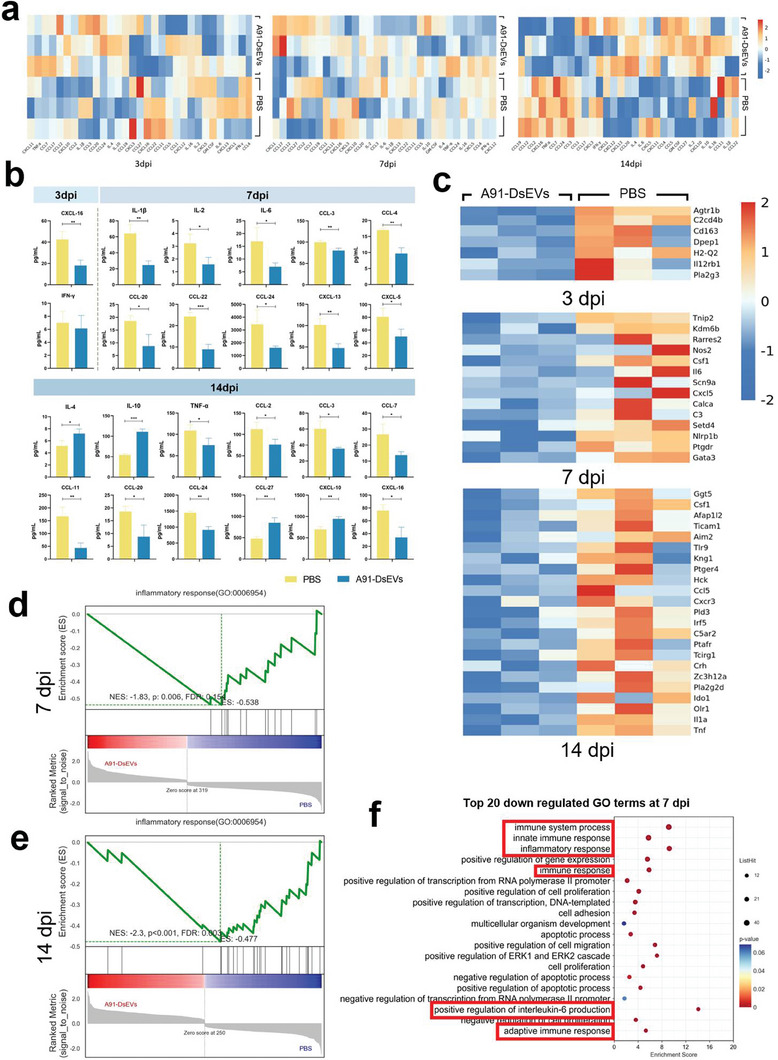
The effect of A91‐DsEVs on the inflammatory signals at the injury site. a) Heatmap to show the Luminex results showing the cytokines level in spinal cord samples at 3, 7, and 14 dpi. b) Bar graph to present the quantitative analysis of the Luminex results, comparing the expression levels of these inflammatory markers at 3, 7, and 14 dpi (*n* = 3). c) Heatmap to illustrate the downregulation of genes associated with inflammation at 3, 7, and 14 dpi (*n* = 3, fold change < 0.5, *p* < 0.05). d,e)  Gene set analysis to demonstrate the downregulation of the inflammatory response at 7 dpi (d) and 14 dpi (e). f) GO enrichment analysis to show the downregulation (top 20 terms displayed) of genes associated with immune and inflammatory responses at 7 dpi. Data were expressed as mean ± SD. (*p < 0.05, **p < 0.01, ***p < 0.001, ****p < 0.0001 as assessed by two‐tailed *t*‐test).

Given the differences in outcomes between treatment with A91‐DsEVs and PBS, we conducted protein level analysis using Luminex to assess the cytokine and chemokine levels in spinal cord samples from the A91‐DsEVs‐treated group and PBS‐treated group at three different time points: 3, 7, and 14 dpi (**Figure** **5**a,b). At 3 dpi, the levels of CXCL‐16 and IFN‐γ were downregulated in the A91‐DsEVs‐treated group. At 7 dpi, reduced levels of IL‐1β, IL‐2, IL‐6, and several other cytokines were detected in the A91‐DsEVs‐treated group. Furthermore, the expression of IL‐4 and IL‐10 increased, while the level of TNF‐α decreased in the A91‐DsEVs‐treated group at 14 dpi. In addition, the expression of other proteins, such as CCL‐3, CCL‐4, CCL‐20, CCL‐24, and CXCL‐10, was diminished in the A91‐DsEVs‐treated group. Notably, the expression levels of CCL‐3, CCL‐20, and CCL‐24 were reduced at both 7 and 14 dpi.

RNA sequencing analysis performed on the injured spinal cord demonstrated that, compared to the PBS treatment, the A91‐DsEVs treatment led to the downregulation of several genes associated with inflammation. The heatmap visualized the downregulated expression of inflammatory‐related genes, including C3, IL‐1α, IL‐6, Clrp1b, and Tnf among others, at 3, 7, and 14 dpi (Figure 5c). Csf‐1, a crucial regulator of macrophage proliferation and migration, showed decreased expression at 7 and 14 dpi. Gene set enrichment analysis revealed a weakened inflammatory response in the A91‐DsEVs‐treated group at 7 (Figure 5d) and 14 dpi (Figure 5e). Furthermore, the Gene ontology (GO) analysis demonstrated the downregulation of genes associated with the immune response and inflammatory processes in the A91‐DsEVs‐treated group at 7 (Figure 5f) and 14 dpi (Figure S8, Supporting Information). In addition, the Kyoto Encyclopedia of Genes and Genomes (KEGG) pathway enrichment analysis exhibited a downregulation of the TNF‐α signaling pathway, JAK‐STAT signaling pathway and IL‐17 signaling pathway in the A91‐DsEVs‐treated group, compared to those in the PBS‐treated group at 7 dpi (Figure S9a, Supporting Information). At 14 dpi, cytokine‐cytokine receptor interactions, the toll‐like receptor signaling pathway, and MAPK signaling pathway were downregulated (Figure S9b, Supporting Information).

These data suggest that A91‐DsEVs attenuate neuroinflammation after SCI, as evidenced by the downregulation of multiple inflammatory signals.

### 2.6 A91‐DsEVs Promoted Axon Regrowth and Neural Survival after SCI

**Figure 6:**
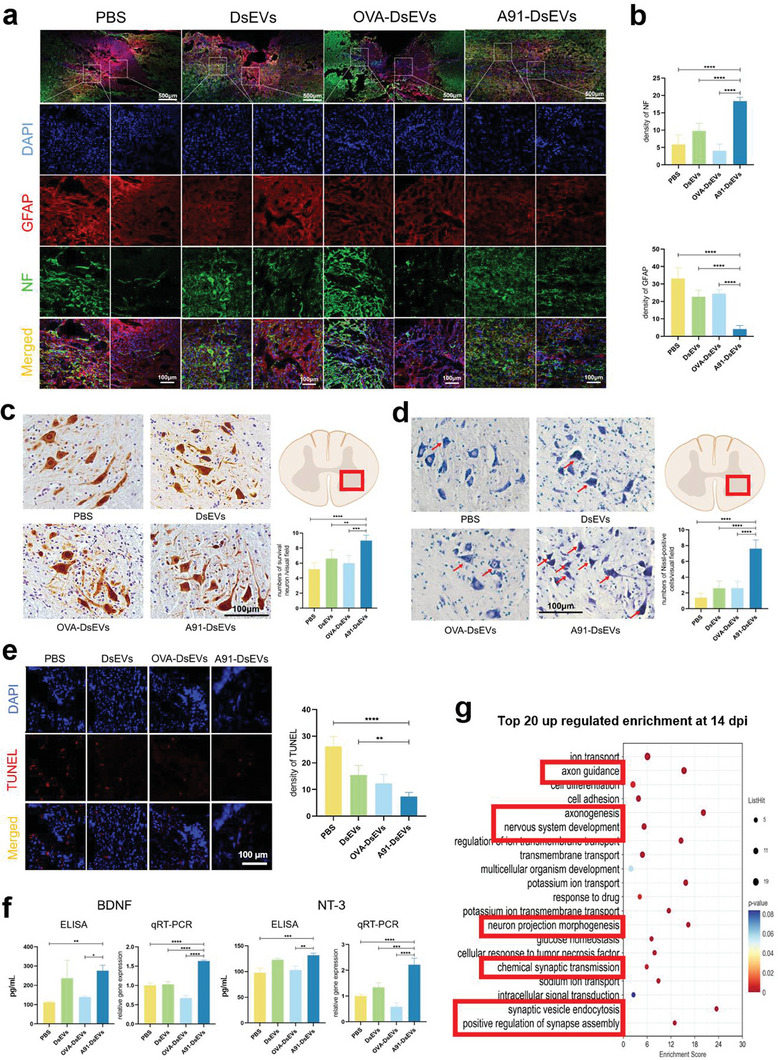
A91‐DsEVs promoted axon regrowth and neuronal survival after SCI. a) Representative immunofluorescence images of NF (green) and GFAP (red) in the spinal cord lesions of mice treated with PBS, DsEVs, OVA‐DsEVs or A91‐DsEVs at 35 dpi (scale bar = 500 µm in low magnification, scale bar = 100 µm in high magnification, *n* = 5). b) A quantitative analysis was performed to determine the mean fluorescence density of NF and GFAP at the center of the injury site (*n* = 5). c) Representative IHC images of NeuN in injured spinal cord from different groups at 35 dpi (Scale bar = 100 µm, *n* = 5) Bar graphs showed the mean numbers of survival neurons per visual field. d) Images of Nissl staining in injured spinal cord from different groups at 35 dpi (Scale bar = 100 µm, *n* = 5). The arrow in (d) points to the Nissl‐positive cells. Bar graphs showed the mean numbers of Nissl‐positive cells per visual field. In (c) and (d), five stained sections were randomly selected from each experimental mouse to evaluate the average number of surviving neurons in the anterior horns of spinal cord. e) Representative TUNEL staining images at 7 dpi (Scale bar = 100 µm, *n* = 5) and quantitative analysis of the fluorescence density (*n* = 5). The density of TUNEL was measured from 5 microscopic fields for each sample. f) The levels of BDNF and NT‐3 detected by ELISA and their gene expression levels detected by qRT‐PCR in the injured spinal cord from different groups at 7 dpi. g) Pathway enrichment analysis of genes that were significantly upregulated (top 20 terms are displayed) in the A91‐DsEVs‐treated group versus the PBS‐treated group at 14 dpi. Data were expressed as mean ± SD. (*p < 0.05, **p < 0.01, ***p < 0.001, ****p < 0.0001 as assessed by One‐way ANOVA with Dunnett's multiple comparisons).

We evaluated the therapeutic effects of A91‐DsEVs. Our findings demonstrated a higher fluorescence density of neurofilament (NF) and a lower fluorescence density of glial fibrillary acidic protein (GFAP) in the A91‐DsEVs‐treated group at 35 dpi (**Figure** **6**a,b). Immunohistochemistry (IHC) staining of neuronal nuclei (NeuN) revealed an increased numbers of surviving neurons in the spinal cord of A91‐DsEVs‐treated group at 35 dpi (Figure 6c). Nissl staining images demonstrated an increase in the number of Nissl‐positive cells in the A91‐DsEVs‐treated group at 35 dpi (Figure 6d). The TUNEL staining results at 7 dpi indicated a reduction in nerve cell apoptosis rate in the A91‐DsEVs‐treated group (Figure 6e). These findings suggest that the immune environment induced by A91‐DsEVs promotes axon regrowth and contributes to neural survival after SCI.

Moreover, we evaluated the expression levels of two major nerve growth factors, NT‐3 and BDNF, using ELISA and quantified their gene expression levels using qRT‐PCR at 7 dpi (Figure 6f). The protein expression levels of NT‐3, BDNF, and their associated genes were higher in the A91‐DsEVs‐treated group than in the comparison groups. Moreover, RNA sequencing analysis at 14 dpi (Figure 6g) revealed an upregulation of genes associated with axon guidance, axonogenesis, nervous system development, neuron projection morphogenesis, chemical synaptic transmission, synaptic vesicle endocytosis, and a positive regulation of synapse assembly in the A91‐DsEVs‐treated group.

### 2.7 A91‐DsEVs Promoted Remyelination after SCI

**Figure 7:**
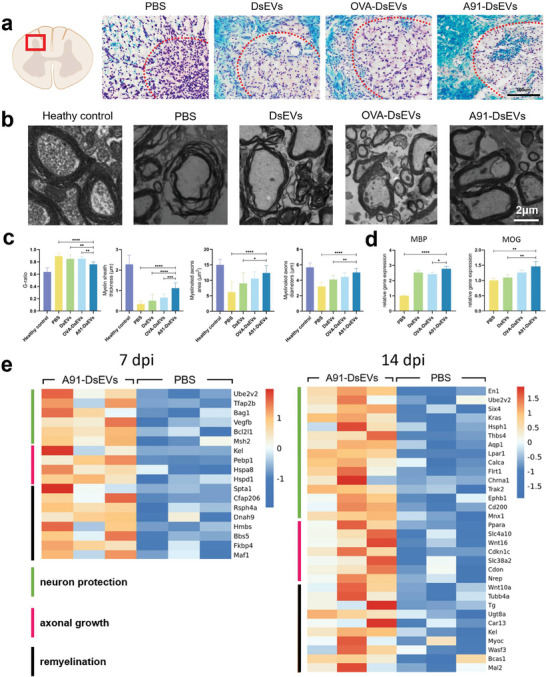
A91‐DsEVs promoted remyelination after SCI. a) Representative LFB staining images of spinal cord tissue from different groups at 35 dpi. The transverse slices of rostral spinal cord segments (5 mm in length, 5 mm far from the injury epicenter) were used for LFB staining. b) Representative images of myelinated axonal regrowth captured by TEM at 35 dpi (Scale bar = 2 µm, *n* = 5). c) Quantitative analysis of the myelination, including G‐ratio, myelin sheath thickness, myelinated axon area, and myelinated axon diameter at 35 dpi (*n* = 5). d) The mRNA expression levels of MBP and MOG measured by qRT‐PCR in the injured spinal cord from different groups at 35 dpi, *n* = 5). e) RNA sequencing results showed the upregulated genes associated with neuron protection, axonal growth and remyelination in the A91‐DsEVs‐treated group versus the PBS‐treated group at 7 and 14 dpi. Data were expressed as mean ± SD. (*p < 0.05, **p < 0.01, ***p < 0.001, ****p < 0.0001 as assessed by One‐way ANOVA with Dunnett's multiple comparisons).

To investigate whether A91‐DsEVs could affect remyelination following SCI, Luxol Fast Blue (LFB) staining and TEM evaluations were performed. LFB staining revealed the highest density of myelin in the spinal cord tissue of the A91‐DsEVs‐treated group at 35 dpi (**Figure** **7**a). TEM analysis was performed at 35 dpi (Figure 7b), and the results were consistent with those of LFB staining. Quantification of the nerve fiber‐specific morphological parameters was conducted, including the G‐ratio, myelinated axon area and diameter, and myelin sheath thickness measurements (Figure 7c). A smaller G‐ratio reflects better remyelination. The G‐ratio of the A91‐DsEVs‐treated group was smaller than those of the other groups. In addition, compared to the OVA‐DsEVs‐treated, DsEVs‐treated, and PBS‐treated groups, the A91‐DsEVs‐treated group showed increased myelinated axon diameters and areas. Furthermore, the mRNA expression levels of MBP and myelin oligodendrocyte glycoprotein (MOG) were assessed. The levels of MBP and MOG mRNA were higher in the A91‐DsEVs‐treated group than in the other groups at 35 dpi (Figure 7d).

RNA sequencing analysis revealed upregulation of numerous genes associated with neuron protection, axonal regrowth, and remyelination in the A91‐DsEVs‐treated group at 7 and 14 dpi (Figure 7e). These data and the results mentioned above (Figure 6) substantiated the hypothesis that A91‐DsEVs treatment could promote neuronal survival and myelination of regenerated axons after SCI.

### 2.8 A91‐DsEVs Improved the Locomotor Function of SCI Model Mice

**Figure 8:**
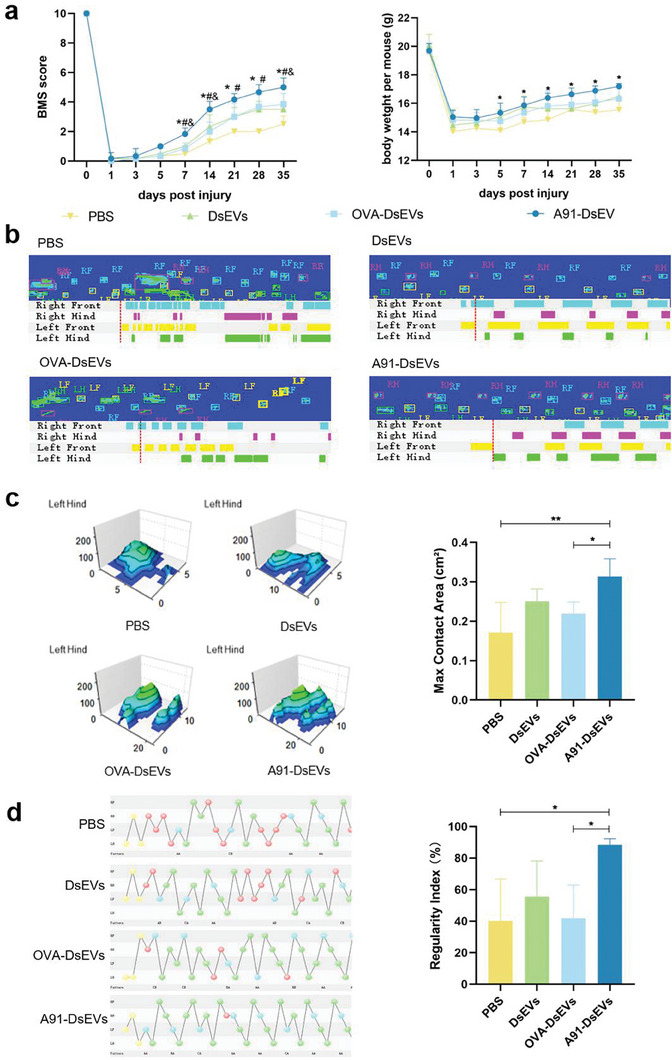
A91‐DsEVs contributed to functional recovery after SCI. a) BMS scores and weight changes of mice from different groups (*p < 0.05, A91‐DsEVs versus PBS treatment; #*p* < 0.05, A91‐DsEVs versus DsEVs treatment; &*p* < 0.05 A91‐DsEVs versus OVA‐DsEVs) (*p < 0.05, **p < 0.01, ***p < 0.001, ****p < 0.0001 as assessed by Two‐way RM ANOVA with Dunnett's multiple comparisons). b) Representative footprint images and time view of mice from different treatment groups at 35 dpi. c) The 3D footprint intensity charts and measurement of maximum contact area of the left hind limb at 35 dpi. d) The footfall patterns graph and regularity index of mice from different groups. Data were expressed as mean ± SD. (*p < 0.05, **p <0 .01, ***p < 0.001, ****p < 0.0001 as assessed by one‐way ANOVA with Dunnett's multiple comparisons).

Recovery of hind limb movement was achieved after A91‐DsEVs treatment at 35 dpi (Figure S10, Supporting Information). We used the Basso mouse score (BMS) to evaluate motor functional recovery in the SCI model mice. All animals exhibited complete hind limb paralysis, with a BMS score of 0 points at 1 dpi. A91‐DsEVs‐treated mice achieved significant functional recovery, with a mean BMS of 5.00±0.63 pionts at 35 dpi. In contrast, the BMS was much lower in the other groups (OVA‐DsEVs: 3.83±0.75, DsEVs: 3.50±0.55, PBS: 2.50±0.55) (**Figure** **8**a). The Catwalk footprint analysis revealed that the A91‐DsEVs‐treated group achieved better hind limb motor function recovery (Figure 8b), with a larger maximum contract area (Figure 8c) and much higher regularity index (improved walking steps) (Figure 8d), compared to those achieved by the other groups.

To assess the biotoxicity of A91‐DsEVs, histopathological examination of the lung, liver, and other major organs in the mice was performed using HE staining at 3 dpi. The results showed normal morphology and structure of the major organs in the A91‐DsEVs‐treated group, without any apparent signs of injury (Figure S11a, Supporting Information). Lactate dehydrogenase (LDH) and alanine aminotransferase (ALT) activity levels were evaluated to determine liver function (Figure S11b, Supporting Information). These findings indicated that the liver function in mice treated with A91‐DsEVs was comparable to those in the other groups. These results suggested that A91‐DsEVs had no significant biotoxicity in mice.

## 3 Discussion

Excessive activated inflammation following CNS injury leads to irreversible dysfunction by disrupting neural circuitry and impairing tissue repair mechanisms.^[^ ^35^ ^]^ Establishing a protective immune microenvironment to inhibit excessively activated inflammation may help promote functional recovery after SCI. Therefore, in this study, we developed a vaccine platform using DsEVs loaded with an APL peptide cargo to induce a protective immune microenvironment at the injury site and facilitate functional recovery after SCI.

Recent studies have emphasized the use of therapeutic vaccines against SCI. DNA‐, RNA‐ and protein‐based vaccines targeting myelin‐associated inhibitors and their associated receptors have been used to treat SCI.^[^ ^36^, ^37^, ^38^ ^]^ Cell‐based vaccination offers a novel approach to the treatment of SCI. As therapeutic vaccines, stem cells promote recovery by inhibiting glial scar expansion and reducing inflammation. However, the immunosuppressive effects of these stem cells may increase tumor risk by suppressing the immune response.^[^ ^39^ ^]^ Antigen‐presenting cells such as dendritic DCs can induce neuroprotection through T cell‐dependent immune responses. This finding suggests the potential of using antigen‐specific DCs to regulate neuroinflammation after SCI.^[^ ^40^ ^]^

Recent studies have highlighted the advantages of EVs as novel therapeutic vaccines. EVs‐based vaccines have several advantages over cell‐based vaccines in terms of their preparation, immunogenicity, storage, safety, and component control.^[^ ^41^ ^]^ Nevertheless, the use of EVs is associated with dispersed distribution and low efficiency, attributed to the following reasons. 1) The non‐specific biodistribution of EVs, leading to their accumulation in unintended organs such as the liver, spleen, lungs, kidneys, and pancreas, poses a significant challenge for their effective utilization. 2) EVs may be taken up by non‐targeted cells.^[^ ^42^ ^]^

The application of DsEVs can enhance the efficacy of immunotherapeutic interventions.^[^ ^43^ ^]^ DsEVs offer the unique ability to directly deliver peptide cargo to lymphoid organs or antigen‐presenting cells where it can activate antigen‐specific T cells, which may help them reach their target.^[^ ^28^ ^]^ Moreover, because DsEVs are rich in MHC II molecules, they can directly upregulate MHC‐II expression on the surface of DCs. In addition, DsEVs as carriers can avoid enzyme degeneration of antigen peptides in the circulation, thereby contributing to the use of a lower concentration of peptide and promoting the efficacy of delivery to DC and T cells.^[^ ^44^ ^]^ Finally, DsEVs follow the endocytic pathway and reach multivesicular endosomes, which are typically targeted for lysosomal degradation after exerting their function.^[^ ^45^ ^]^ This represents a possible fate for DsEVs after internalization and completion of their functional effects.

MBP_87‐99_A^91^ is an APL derived from a specific segment (amino acids 87–99) of MBP, in which the lysine residue at position 91 is replaced with alanine. This site has pivotal significance in mediating TCR interactions, as validated by previous studies.^[^ ^46^ ^]^ Replacing lysine with alanine in the peptide sequence modulates TCR‐binding kinetics.^[^ ^47^, ^48^ ^]^ As a partial TCR agonist, MBP_87‐99_A^91^ downregulates the production of pro‐inflammatory cytokines, increases the levels of Th2 cytokines, and generates a microenvironment with anti‐inflammatory features.^[^ ^49^ ^]^ In our experiment, DsEVs were loaded with MBP_87‐99_A^91^ peptide. These A91‐DsEVs, containing MHC II and costimulatory molecules, presented the MBP_87‐99_A^91^ peptide‐MHC II complex directly to CD4^+^ T cells, thereby inducing polarization of CD4^+^ T cells toward the Th2 phenotype.

After SCI, the local tissue structures are disrupted, leading to antigen exposure at the site.^[^ ^50^ ^]^ MBP, a crucial constituent of the myelin sheath, is frequently exposed after SCI and triggers immune cell activity.^[^ ^51^ ^]^ A91‐DsEVs stimulated the conversion of CD4^+^ T cells into antigen‐specific Th2 and Treg cells, enabling their recruitment to injury sites. CCR4 is a pivotal chemokine receptor that plays a significant role in the recruitment of both Th2 and Treg cells.^[^ ^52^ ^]^ Studies have demonstrated that increased CCR4 expression in Treg cells promotes their migration to inflammatory sites and exerts immunosuppressive effects.^[^ ^53^, ^54^ ^]^ Moreover, a recent study indicated that CCR4 antagonists can selectively inhibit the migration of Th2 cells to inflamed tissues by blocking highly expressed CCR4 receptors on these cells.^[^ ^55^ ^]^ In our study, the upregulation of CCR4 expression in antigen‐specific T cells, stimulated by A91‐DsEVs, suggests that CCR4 is involved in regulating the recruitment of Th2 and Treg cells to the injury site.

DsEVs are non‐immunogenic or possess very low immunogenicity and tend to be well‐tolerated by the immune system.^[^ ^41^ ^]^ In fact, MBP_87‐99_A^91^ used herein has also been shown to be a non‐immunogenic peptide that can mediate the immune response under pathological conditions, and change the polarity of inflammatory T cells, without inducing immunogenicity.^[^ ^56^, ^57^, ^58^, ^59^ ^]^

To load the peptide onto DsEVs, we used an indirect method involving antigen feeding into DCs and subsequent collection of the supernatant for DsEVs isolation. This loading strategy ensures a favorable interaction between DsEVs and T cells, as it guarantees the association of the peptide with major MHC molecules and, as a result, fosters peptide presentation and T‐cell activation.^[^ ^60^, ^61^ ^]^ To enhance the loading efficiency of the MBP_87‐99_A^91^ peptide in DsEVs, we fused it to CPP. CPPs are peptides that facilitate the delivery of cargo molecules into the cytosol. The CPP‐peptide cargo is delivered to the cytosol by direct interactions with the components of the cell surface or the plasma membrane. TAT_47‐57_, a CPP derived from HIV TAT protein, is one of the most commonly used CPPs for intracellular peptide delivery.^[^ ^62^ ^‐^ ^6‐4^ ^]^ Previous studies have demonstrated that TAT_47‐57_‐linked peptide cargos possess the increased ability to translocate intracellularly into DCs and extend the presentation of MHC‐peptide complexes to antigen‐specific T cells, facilitating enhanced immune responses without causing biotoxicity.^[^ ^65^, ^66^ ^]^ Therefore, in this study, we used the TAT_47‐57_‐linked peptide to upregulate the loading amount of MBP_87‐99_A^91^ in DC, which contributed to promoting the loading efficiency of the MBP_87‐99_A^91^ peptide in DsEVs.

The balance between different subtypes of CD4^+^ T cells is crucial for neuroprotection.^[^ ^67^ ^]^ In our study, we demonstrated that immunization with A91‐DsEVs significantly upregulated the levels of Th2 and Treg cells and downregulated the level of Th1 and Th17 cells in vivo. The Th1 and Th17 cells primarily induce spinal cord inflammation, causing tissue damage. IFN‐γ is a prototypical Th1 cytokine. The continuous expression of Th1 cytokines at the lesion site may lead to neuronal damage and demyelination.^[^ ^7^ ^]^ They are also capable of activating M1 macrophages and aggravating lesions in SCI.^[^ ^4^ ^]^ Th17 produces cytokines (e.g., IL‐17A, IL‐6, and IFN‐γ), leading to the abnormal neuroinflammatory response including the excessive activation of microglia and the recruitment of other immune cell types, which are involved in neuron or axon injury and demyelination.^[^ ^68^, ^69^ ^]^

IL‐4 is a Th2‐cell‐type cytokine that protects against infection and promotes tissue regeneration. The transition from the Th1 to Th2 response is dominated by anti‐inflammatory factors, which create a beneficial microenvironment, induce the polarization of microglia and macrophages, and facilitate the protection of neurons.^[^ ^70^ ^]^ However, the presence of Th2 cells in the CNS may be associated with certain risks. Therefore, the protective role of IL‐4 may be detrimental to the developing brain.^[^ ^71^ ^]^ Previous studies have demonstrated that neuroimmune responses in fetuses and newborns markedly differ from those observed in adults.^[^ ^71^, ^72^ ^]^ Moreover, a pathogenic Th2 cell subset revealed by single‐cell RNA ‐seq may be correlated with disease severity.^[^ ^73^ ^]^ In general, Th2 cells play a positive role.^[^ ^74^ ^]^ In fact, Th2 cells have been shown to promote recovery in SCI and other neurological disorders by secreting protective cytokines.^[^ ^8^, ^9^, ^10^ ^]^

Treg cells can inhibit inflammatory response and are thought to be targets for balancing the immune environment and improving functional outcomes in CNS trauma.^[^ ^75^ ^]^ It has been shown that Treg cell‐derived cytokines such as TGF‐β and osteopontin may help maintain immune homeostasis by suppressing excessive immune responses.^[^ ^76^, ^77^ ^]^ IL‐10 is a key immunesuppressive cytokine produced by regulatory T‐cells.^[^ ^78^ ^]^ Accumulating evidence has demonstrated that Tregs alleviate neural injury, promote neurogenesis and protect the blood‐brain barrier via IL‐10.^[^ ^79^ ^]^ Studies have also shown that an increased level of IL‐10 can induce an M2 subtype bias in macrophages/microglia. Tregs mediate the differentiation of macrophages toward an M2‐like state by increasing the levels of the inflammation‐dampening cytokines under inflammatory conditions.^[^ ^80^, ^81^ ^]^

The polarization state of macrophages/microglia are regulated by Th2 or Treg cells.^[^ ^81^ ^]^ Therefore, the increased number of M2‐polarized macrophages/microglia induced by A91‐DsEVs may be attributed to polarized T cells. A series of studies has shown that the microenvironment established by M2‐polarized macrophages/microglia may release nerve growth factors and promote immune protection and axonal growth in SCI.^[^ ^34^ ^]^ In addition, the multiple downregulated pro‐inflammatory cytokines, such as IL‐6, TNF‐α, IL‐1β, suggested that A91‐DsEVs effectively established a neuroprotective immune environment and inhibited excessive activated inflammatory response after SCI.

The expression of IL‐4 and IL‐10 at the injury site was elevated at both 7 and 14 dpi following the A91‐DsEVs injection. These findings suggested a sustained effect of A91‐DsEVs treatment. The expression levels of CXCL‐16 and IFN‐γ were downregulated in A91‐DsEVs‐treated group at 3 dpi, suggesting that A91‐DsEVs may inhibit neuroinflammation in the early phase. This finding may be attributed to the activation and infiltration of peripheral DCs and CD4^+^ T cells into the injured area, following injection of A91‐DsEVs, thereby regulating the immune response. CXCL‐16 is an inflammatory cytokine associated with early stage neutrophil activation; its levels could be increased by the release of pro‐inflammatory cytokines such as IFN‐γ.^[^ ^82^ ^]^

Our results also showed that CCL‐3, CCL‐20, and CCL‐24 levels were decreased in the A91‐DsEVs‐treated group. CCL‐3 has been shown to be upregulated by the MAPK and NF‐κB signal pathways.^[^ ^83^ ^]^ CCL‐20 may aggravate neuroinflammation via Th17 cell recruitment.^[^ ^84^ ^]^ CCL‐24 is a chemokine that promotes immune cell trafficking and regulates inflammatory responses. Treatment with an anti‐CCL‐24 monoclonal antibody could exert an immunosuppressive effect.^[^ ^85^ ^]^ The levels of these three cytokines were decreased in the A91‐DsEVs‐treated group at 7 and 14 dpi. These results provide further evidence that A91‐DsEVs may induce a protective immune response after CNS trauma.

## 4 Conclusion

This study demonstrated that A91‐DsEVs induce antigen‐specific CD4^+^ T cells homing to the injury site, establish a protective immune microenvironment, and promoted functional motor recovery in the SCI mice. Our results suggest that DsEVs vaccination strategy may help treat CNS truama or inflammatory diseases.

## 5 Experimental Section

### APLs

Peptide synthesis grade reagents were purchased from QYAOBIO (China). The peptide sequence for MBP_87‐99_A^91^ was VHFFANIVTPRTP. The peptide sequence for a specific segment of OVA, consisting of amino acids 323–339, was ISQAVHAAHAEINEAGR. The peptide sequence for TAT_47‐57_ was YGRKKRRQRRR. Prior to final deprotection and cleavage, the MBP_87‐99_A^91^ peptide segment was labeled with fluorescein FITC (5‐FITC‐(Acp)‐VHFFANIVTPRTP‐YGRKKRRQRRR). The peptides were purified using high‐performance liquid chromatography, resulting in a purity exceeding 95%.

### Animals

Six‐eight‐week‐old C57BL/6 female mice were kept in a 12‐h light/dark cycle at 21±2 ˚C and 50% humidity, and they were allowed free access to food and water. Female mice were chosen for the whole experiments due to fewer post‐operative complications and easier manual bladder expressions after experimental paralysis compared to male mice. In addition, no sex differences were observed in lesion size or locomotor recovery after SCI in mice.^[^ ^86^ ^]^ This study received approval from the Ethics Committee (no. SYDW2019–111) of the Second Affiliated Hospital of Harbin Medical University (Harbin, China) in accordance with Chinese experimental animal legislation. All the authors compliance with all relevant ethical regulations.

### Cell culture

DCs were generated through the culture of bone marrow cells isolated from femurs of C57BL/6 mice (six‐eight‐week‐old) in complete medium (Gibco, USA) as previously described.^[^ ^87^ ^]^ Cells were cultured in complete medium added with murine GM‐CSF and IL‐4 (20 ng mL^−1^, Pepro Tech, USA) and refreshed every two days. On day 6, the cells were harvested for further analysis. Next, the medium was supplemented with TAT_47‐57_‐MBP_87‐99_A^91^ or TAT_47‐57_‐ OVA antigen (1 mg mL^−1^). After 24 h, exosome‐free complete medium was used. After 24 h of incubation, the supernatants were collected for further analysis.

### DsEVs Isolation

DsEVs isolation was performed as previously described.^[^ ^88^, ^90^ ^]^ As briefly, the supernatant was purified by sequential centrifugation (4˚C, 500 ***g*** for 5 min; 2000 ***g*** for 20 min; 10,000 ***g*** for 30 min) to remove cells and debris. Subsequently, the purified supernatant underwent 0.2 µm filtration and ultra‐centrifugation for further refinement (4˚C, 120,000 ***g*** for 90 min; Himac CP100NX ultracentrifuge, Hitachi Koki, Japan). The pellet was then washed in PBS and ultra‐centrifugated again (4˚C, 120,000 ***g*** for 90 min). Finally, The pellet was resuspended in PBS and stored at −80˚C until use.^[^ ^91^ ^]^

### DsEVs Characterization

*Western blotting*: Primary antibodies: anti‐CD63 (ab217345, Abcam, USA), anti‐Alix (ab275377, Abcam), anti‐TSG101 (ab125011, Abcam), anti‐Calnexin (ab313243, Abcam). The membranes were developed using ECL kit (Yeasen, China).

*TEM*: DsEVs sample was examined by TEM (Hitachi TEM system, Japan).

*NTA*: 10 µL DsEVs suspension was loaded into the sample chamber of ZetaVIEW S/N 252 (Particle Metrix GmbH, Germany). Data analysis was performed with software (ZetaView 8.04.02, Germany).

*Detection of the loading rate of TAT_47‐57_‐MBP_87‐99_A^91^ into DsEVs*: After co‐culturing the FITC‐labeled peptide with DCs for 24 h, DCs were collected and stained with phalloidin (Actin‐Tracker Red‐594, C2205S, Beyotime) and 4′,6‐diamidino‐2‐phenylindole (DAPI, C1005, Beyotime, China). After the isolation of DsEVs.

*DsEVs internalized by DCs and CD4^+^ T cells in vitro*: DsEVs were labelled with the Dil (Solarbio life sciences, China) and then co‐cultured with DCs or CD4^+^ T cells for 24 h. Cells were fixed and followed by staining with phalloidin (Actin‐Tracker Green‐488, C2201S, Beyotime) and DAPI (C1005, Beyotime). The images were obtained by scanning confocal fluorescence microscopy.

### MLR

DCs were generated as mentioned above. A total of 1 × 10^4^ DCs were co‐cultured with 1 × 10^5^ splenic T cells. A91‐DsEVs, OVA‐DsEVs, DsEVs or the same amount of PBS were added in the MLR in different groups.

### CCK‐8 Assay

The cell concentration to 5000 mL was first adjusted. Cell suspension was treated by different DsEVs or PBS and 100 µL of cell suspension was added to each well of a 96‐well plate. On day 3 and day 5 of MLR culture, the culture medium was replaced and added with 10 µL of CCK‐8 solution. Following a 2‐h inubation, the absorbance at 450 nm was measured using a microplate reader (Bio Rad iMak, Japan).

### SCI Model

The surgery procedures were the same as described elsewhere.^[^ ^92^ ^]^ Briefly, the mice were divided randomly into four groups. Under general anesthesia (1.25% 2,2,2‐tribromoethanol, 0.02 Ml g^−1^, Nanjing Aibei Biotechnology, China) and sterile conditions, T10 spinal cord segment was exposed by laminectomy. Then a weight‐drop device was utilized to induce spinal cord contusion injury (RWD Life Science Corp, China). A 5.0 g rod was dropped from a height of 5.0 cm, striking the exposed spinal cord. Following the impact, the muscle and skin layers were meticulously sutured in sequential order. Two hours after SCI, mice were injected with 100 µg A91‐DsEVs, OVA‐DsEVs, DsEVs dissolved in 100 µL PBS or the same amount of PBS via the tail vein.

### In Vivo Tracing Analysis

First, A91‐DsEVs, OVA‐DsEVs, and DsEVs using DiR dye was labeled according to the manufacturer's instructions. The specific procedure was as follows: 10 µL of DiR working solution was added to 100 µL of each vesicle group, and they were incubated at 37 °C for 30 min. Subsequently, excess dye was removed by washing. These DiR‐labeled vesicles were then injected into the spinal cord‐injured mice via tail vein injection (100 µL mouse^−1^). After 6 h, various organs including the heart, liver, spleen, lungs, kidneys, and spinal cord were collected from each group of mice, and the distribution of DiR fluorescence was observed using the Living Image software (version 4.4, USA).

The splenic CD4^+^ T cells were isolated using the mouse CD4^+^ T cell isolation kit (Miltenyi Biotec, Germany) at 3 dpi and then stained with Dil. Dil‐labeled CD4^+^ T cells were then injected into SCI mice through peripheral injection (tail vein, 3×10^6^ in 100 µL PBS). After 24 h, the biodistribution of Dil‐labeled CD4^+^ T cells was photographed using an in vivo imaging system (IVIS, Night OWL II LB983, Germany), including fluorescence images of the liver, heart, spleen, lung, kidney, and spinal cord. Bruker MI SE software was used for image analysis.

### ELISA

The CIITA expression level of DCs was detected by ELISA kit (JingKang Bio, China) after co‐culturing with different groups of DsEVs or PBS for 24 h. The expression level of IL‐4, IL‐10, IFN‐γ, IL‐17A, NT‐3, BDNF in supernatant of MLR and spinal cord tissue were detected by ELISA kit (JingKang Bio, China). After sacrificing SCI mice at 7 dpi, their spinal cords were rapidly collected and stored at −80 °C for protein isolation. Segments of injured spinal cord (10 mm in length) were homogenized in RIPA buffer. The samples were maintained on ice for 30 min with intermittent vortexing every 5 min, followed by sonication in three pulse bursts for 10 s and repeat vortexing. Then the samples were then centrifuged at 14,000 ***g*** at 4 °C for 15 min and the supernatant for the ELISA was collected. Protein concentration in the supernatant was quantified using BCA assay. The absorbance was measured with a microplate reader at 450 nm (Bio Rad iMak, Japan).

### Tissue Processing

The whole segments (10 mm in length, centered on the injury epicenter) and the rostral spinal cord segments (5 mm in length, 5 mm far from the injury epicenter) were separated and post‐fixed in paraformaldehyde overnight at 4 ˚C. The whole segments were washed dehydrated in a gradient sucrose solution (10%, 20%, and 30%). Samples were frozen and sectioned into 5 µm‐thick transverse and longitudinal sections. The rostral spinal cord segments were washed, embedded in paraffin wax, and subsequently sectioned into transverse slices with a thickness of 5 µm.

### Immunofluorescence

Sample sections or cells were incubated with primary antibodies against IFN‐γ (ab280353, Abcam), IL‐4 (bs‐0581R, Bioss antibodies), IL‐17A (1:200, ab302922, Abcam), Foxp3 (12653S, Cell Signaling Technology, USA), CD4 ( 67786‐1‐Ig, Proteintech, China), iNOS (22226‐1‐AP, Proteintech, China), CD206 (24595S, Cell Signaling Technology), F4/80 (71299S, Cell Signaling Technology, USA), Iba‐1 (ab283346, Abcam), NF (2837S, Cell Signaling Technology), GFAP (60190‐1‐Ig, Proteintech) overnight at 4 ˚C. After incubation with the secondary antibodies including Goat anti‐rat IgG(H+L) Alexa Fluor 647 (ab150159, Abcam), Goat anti‐mouse IgG(H+L) Coralite 594 (SA00013‐3, Proteintech) and Goat anti‐rabbit IgG(H+L) FITC (SA00003‐2, Proteintech) for 2 h in dark, sections were washed with PBS, and the nuclei were counterstained with DAPI (Beyotime). Fluorescent images were captured using a confocal laser scanning microscope (Zeiss LSM800, Germany). The numbers of CD4^+^ T cells and different subsets of T cell were achieved by were quantified from 5 microscopic fields around the injured site for each sample. The numbers of iNOS^+^F4/80^+^ cells, CD206^+^F4/80^+^ cells, F4/80^+^ cells, iNOS^+^Iba‐1^+^ cells, CD206^+^Iba‐1^+^ cells and Iba‐1^+^ cells were randomly counted from 5 microscopic fields in the injured site for each sample.

### TUNEL Staining

The transverse sections of rostral spinal cord segments (5 mm in length, 5 mm far from the injury epicenter, 5 µm in thickness) were used for TUNEL staining according to the manufacturer's protocol. The fluorescent images were captured using a confocal laser scanning microcopy (Zeiss LSM800).

### IHC

The transverse slices of rostral spinal cord segments (5 mm in length, 5 mm far from the injury epicenter) were used for IHC. After dewaxed, rehydrated and antigen retrieval, the tissues were blocked, then incubated with a rabbit anti‐NeuN antibody (24307S, Cell Signaling Technology) at 37 ˚C for 2 h and goat anti‐rabbit IgG secondary antibody (1:100; Zhongshan Jinqiao, China), followed by DAB staining (Zhongshan Jinqiao). Five stained sections were randomly selected from each experimental mouse to evaluate the average number of surviving neurons in spinal cord.

### LFB Staining

The transverse slices of rostral spinal cord segments (5 mm in length, 5 mm far from the injury epicenter) were used for LFB staining. A series of sections were dewaxed to 95% ethanol and then soaked in LFB (Solarbio Life Science, China) and then washed with 95% ethanol. Then the sections were differentiated by LFB differentiation solution (Solarbio Life Science) for 15s and in 70% ethanol for 30s. After rinsed in distilled water, sections were observed under the microscope until the gray and white matter was clear. Then crystal violet staining solution (Solarbio Life Science) were used for re‐dyeing the section. After washed with water, sections were dehydrated and then sealed with neutral gum.

### Nissl Staining

The transverse slices of rostral spinal cord segments (5 mm in length, 5 mm far from the injury epicenter) were used for Nissl staining. A series of sections were dewaxed to 80% ethanol and then treated with Nissl dye (Solarbio Life Science) (56 ˚C, 1.5 h), then washed, differentiated in 95% alcohol, dehydrated and then sealed. Five stained sections were randomly selected from each experimental mouse to evaluate the average number of Nissl positive cells.

### TEM Experiments

The whole segments (5 mm in length, centered on the injury epicenter) of spinal cords were fixed in 2.5% glutaraldehyde for 24 h at 4 ˚C, followed by transferred into 2% osmic acid for 2 h. Then the tissues were embedded after dehydration. After solidification, the spinal cord segments were cross sectioned (50 nm thickness). The thick sections were stained with 0.5% toluidine blue and imaged using a transmission electron microscopy (Hitachi TEM System, Japan).

### Flow Cytometry

For cell surface marker staining, cells were stained with antibody on ice for 30 min. For intracellular staining, cells were fixed and permeabilized by Intracellular Fixation & Permeabilization Buffer Set (Thermofisher Scientific, USA) for 30 min, followed by antibody incubation recommended concentration for 30 min. Finally, data was acquired using Apogee flow system (A60‐Universal, UK) and FlowJo_v10.8.1 software. The following fluorochrome‐labeled antibodies and staining kits were used for flow cytometry according to the manufacturers’ protocols: anti‐mouse‐CD3 FITC (45‐0031‐82, Thermofisher Scientific, USA), anti‐mouse‐CD3 PerCP‐Cyanine5.5 (11‐0031‐82, Thermofisher Scientific), anti‐mouse‐CD4 PE (12‐0041‐82, Thermofisher Scientific), anti‐mouse‐ IFN‐γ FITC (Cat# 11‐7311‐82, Thermofisher scientific, USA), anti‐mouse‐ IL‐4 APC (Cat# 17‐7041‐82, Thermofisher Scientific), anti‐mouse‐ IL‐17A PerCP‐Cyanine5.5 (12‐0041‐82, Thermofisher Scientific), mouse regulatory T cell staining kit (88–8111, Thermofisher Scientific), Rat IgG2a kappa Isotype Control (eBR2a) PerCP‐Cyanine5.5 (45‐4321‐80, Thermofisher Scientific), Rat IgG1 kappa Isotype Control (eBRG1) APC (17‐4301‐81, Thermofisher Scientific), Rat IgG1 kappa Isotype Control (eBRG1) FITC (11‐4301‐81, Thermofisher Scientific), Rat IgG2a Anti‐Mouse control PE (88‐8111‐40, Thermofisher Scientific).

### RNA Isolation and qRT‐PCR

DCs were collected for RNA isolation and qRT‐PCR after stimulated by different DsEVs or PBS for 24 h to assess the gene expression levels of CIITA, RFX5, RFXAP, and RFXANK. Additionally, spleen samples from SCI mice were collected after 3 days of injecting different DsEVs or PBS. These splenic CD4^+^ T cells were then utilized for RNA isolation and subsequent qRT‐PCR analysis. As for RNA isolation and qRT‐PCR of spinal cord issue, 10 mm segments of spinal cord (5 mm on each side of the injury epicenter) were isolated. After the extraction of total RNA, cDNA was synthesized. A Stratagene Mx3005p real‐time PCR system (Agilent Technologies, USA) was used for detection. The primer sequences used were provided in Table S1 (Supporting Information).

### Library Preparation and RNA Sequencing Analysis

The libraries construction and transcriptome analysis were conducted by OE Biotech Co., Ltd. (Shanghai, China). Differential expression analysis was performed using the DESeq25. *P*‐value < 0.05 and foldchange > 2 or foldchange < 0.5 was set as the threshold for significantly differential expression gene (DEGs). GO analysis and KEGG pathway enrichment of DEGs were performed respectively.

### BMS

The BMS primary scoring system assesses hind limb motor function on a scale of 0 (complete paralysis) to 9 (completely normal). Mice were observed for 5 min on a flat surface, and hind limb motor function was assessed using the single‐blind method by two independent blind observers. The average score given by both observers for each hind limb was recorded as the BMS score for the sample.

### Catwalk‐Assisted gait analysis

Catwalk‐assisted gait analysis (Catwalk XT, Noldus, Netherlands) was used to further detect the motor function of SCI mice.

### Drug Toxicity Test of DsEVs to Major Organs

After 3 days of DsEVs injection, heart, liver, spleen, lung, and kidneys were taken out for hematoxylin‐eosin staining (HE staining). The series of tissue sections were immersed in xylene, rehydrated through decreasing concentration of ethanol solution, rinsed with distilled water, and then stained with hematoxylin for 2 min. After stained with eosin for 1 min, the sections were dehydrated and sealed.

Simultaneously, the activities of LDH and ALT were measured by the enzyme labeling method using the enzyme assay kits (Wanlei Bio, China). The absorbance OD value was measured with a microplate reader (Bio Rad iMak, Japan).

### Statistical Analysis

SPSS 24.0 and GraphPad Prism 9.0 software were used for statistical analysis and the plotting of statistical graphs. All experimental results were expressed as mean ± SD. The *t*‐test was used for two‐group comparisons. One‐way ANOVA was used to compare more than two groups. Two‐way RM ANOVA was used to compare BMS and body weight change in different groups. Statistical significance was accepted for values of *p* < 0.05 (*p < 0.05, **p < 0.01, ***p < 0.001, ****p < 0.0001).

### Ethics Approval Statement

This study received approval from the Ethics Committee (no. SYDW2019–111) of the Second Affiliated Hospital of Harbin Medical University (Harbin, China) in accordance with Chinese experimental animal legislation. All the authors compliance with all relevant ethical regulations.

## Conflict of Interest

The authors declare no conflict of interest.

## Supporting information

Supporting Information

Supplementary Table S1

## Data Availability

The data that support the findings of this study are available from the corresponding author upon reasonable request.
