# Supplementary material for: Small Extracellular Vesicles Derived from Altered Peptide Ligand‐Loaded Dendritic Cell Act as A Therapeutic Vaccine for Spinal Cord Injury Through Eliciting CD4+ T cell‐Mediated Neuroprotective Immunity
Source: Adv Sci (Weinh). 2023 Nov 30;11(3):2304648. doi: 10.1002/advs.202304648 (PMC10797491; doi:10.1002/advs.202304648)
Supplement: Supplementary file 1 — Supporting Information [file ADVS-11-2304648-s001.pdf]

ADVANCED SCIENCE
Open Access

Supporting Information

for *Adv. Sci.*, DOI 10.1002/advs.202304648

Small Extracellular Vesicles Derived from Altered Peptide Ligand-Loaded Dendritic Cell Act as A Therapeutic Vaccine for Spinal Cord Injury Through Eliciting CD4$^+$ T cell-Mediated Neuroprotective Immunity

Sikai Wang, Guanglei Li, Xiongjie Liang, Zexuan Wu, Chao Chen, Fawang Zhang, Jiawen Niu, Xuefeng Li, Jinglong Yan, Nanxiang Wang, Jing Li and Yufu Wang**

Supporting Information

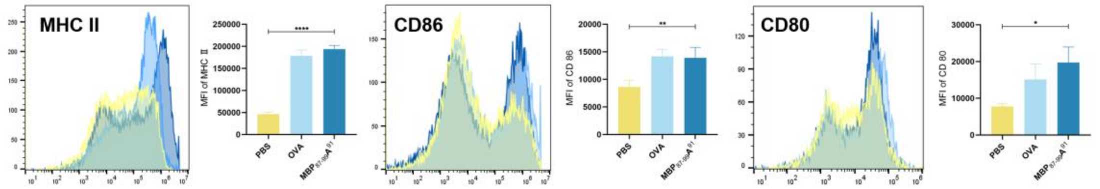

Figure S1. The expression of MHC II and costimulatory molecules in DCs after loading with $MBP_{87\text{-}99}A^{91}$. Data were expressed as mean ± SD. (* p<0.05, ** p<0.01, *** p<0.001, **** p<0.0001 as assessed by One-way ANOVA with Dunnett's multiple comparisons).

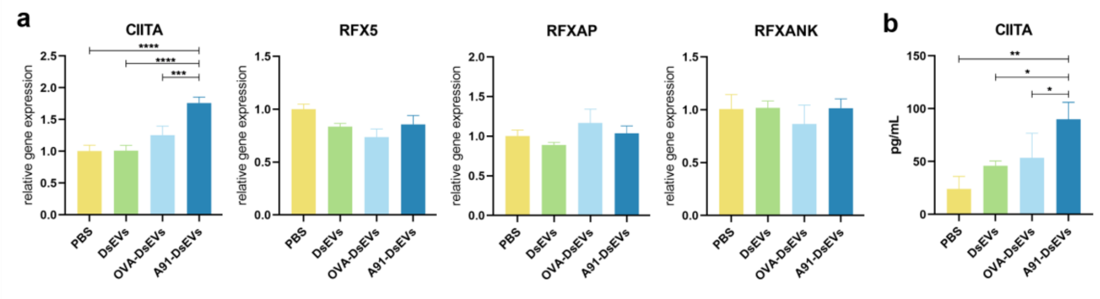

Figure S2. A91-DsEVs regulated the levels of key molecules regulating MHC-II expression on DCs. (a) qRT-PCR results showed a significant increase in CIITA gene expression in DCs treated with A91-DsEVs. (b) The ELISA result demonstrated a rise in CIITA protein levels following A91-DsEVs treatment (n=3). Data were expressed as mean ± SD. (* p<0.05, ** p<0.01, *** p<0.001, **** p<0.0001 as assessed by One-way ANOVA with Dunnett's multiple comparisons).

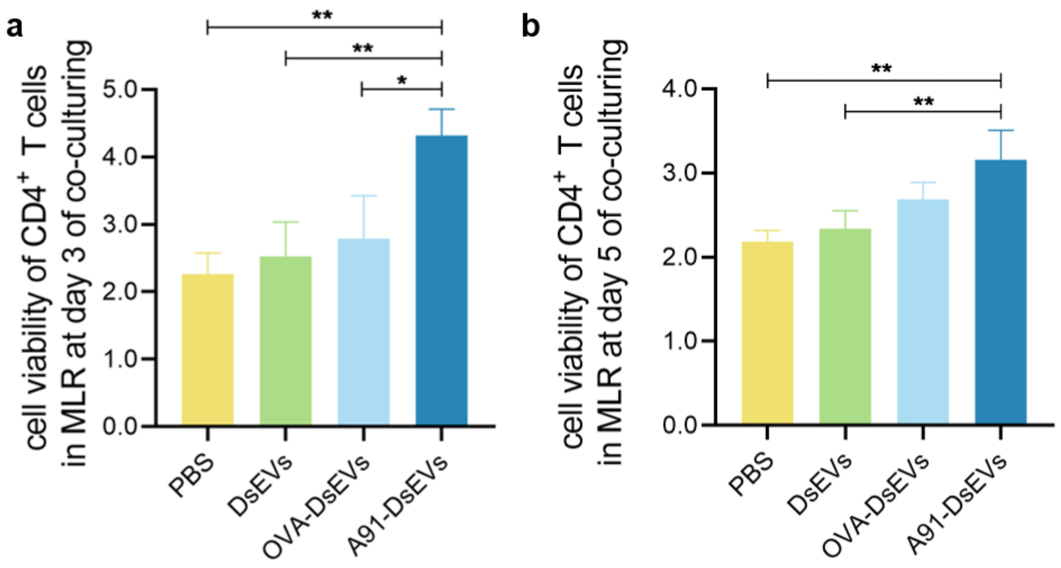

Figure S3. A91-DsEVs promoted the proliferative response of CD4^{+} T cells on day 5 after MLR. The CCK-8 assay revealed enhanced viability of CD4^{+} T cells in the A91-DsEVs treated group compared to the other three groups (n=3). Data were expressed as mean ± SD. (* p<0.05, ** p<0.01, *** p<0.001, **** p<0.0001 as assessed by One-way ANOVA with Dunnett's multiple comparisons).

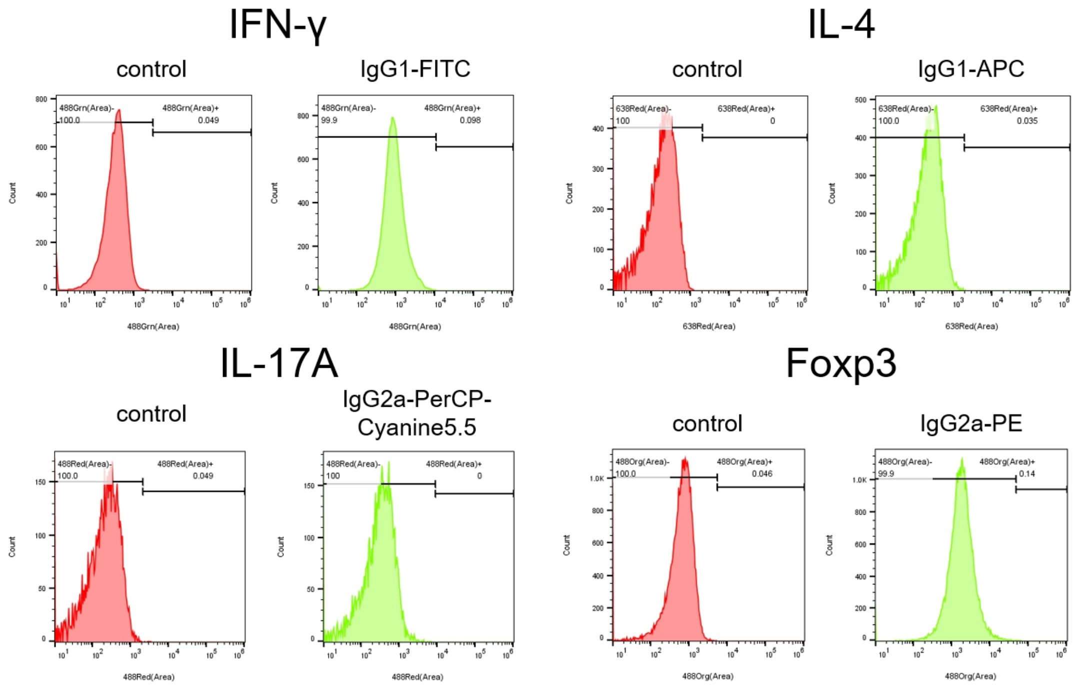

Figure S4. The isotype used for flow cytometry. The images showed isotype of IFN-γ, IL-4, IL-17A, and Foxp3 fluorescence antibody used for flow cytometry.

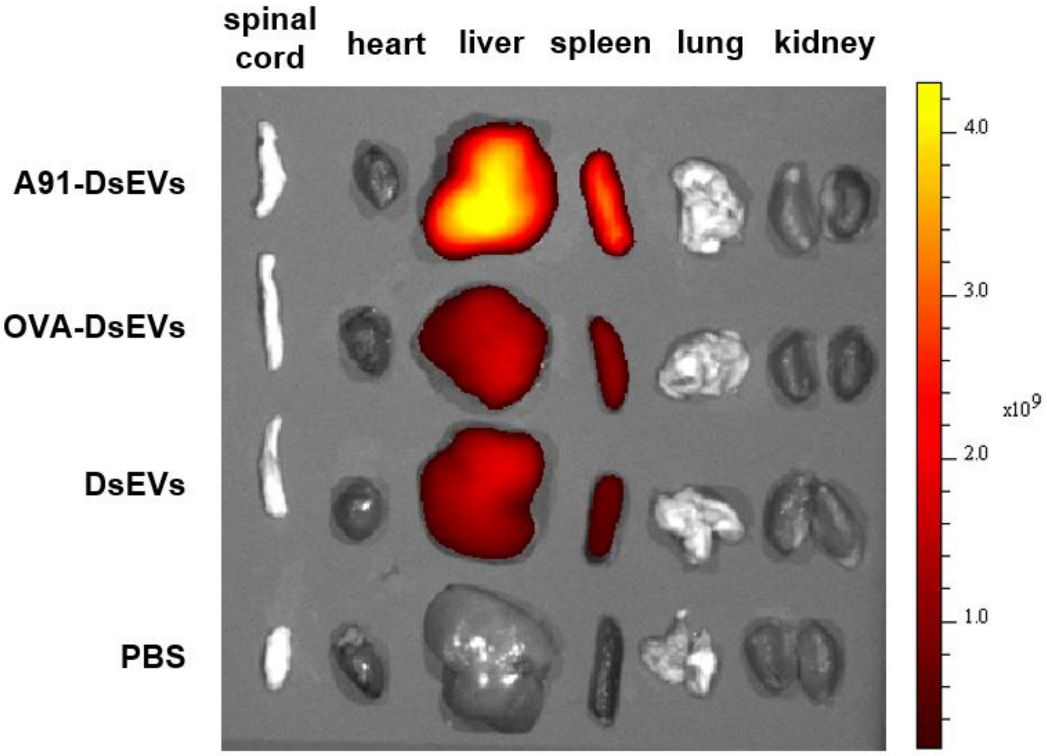

Figure S5. In vivo tracing of different DsEVs in SCI mice. The images showed the distribution of DiR-labeled different DsEVs in SCI mice after 6 hours of injection (n=3).

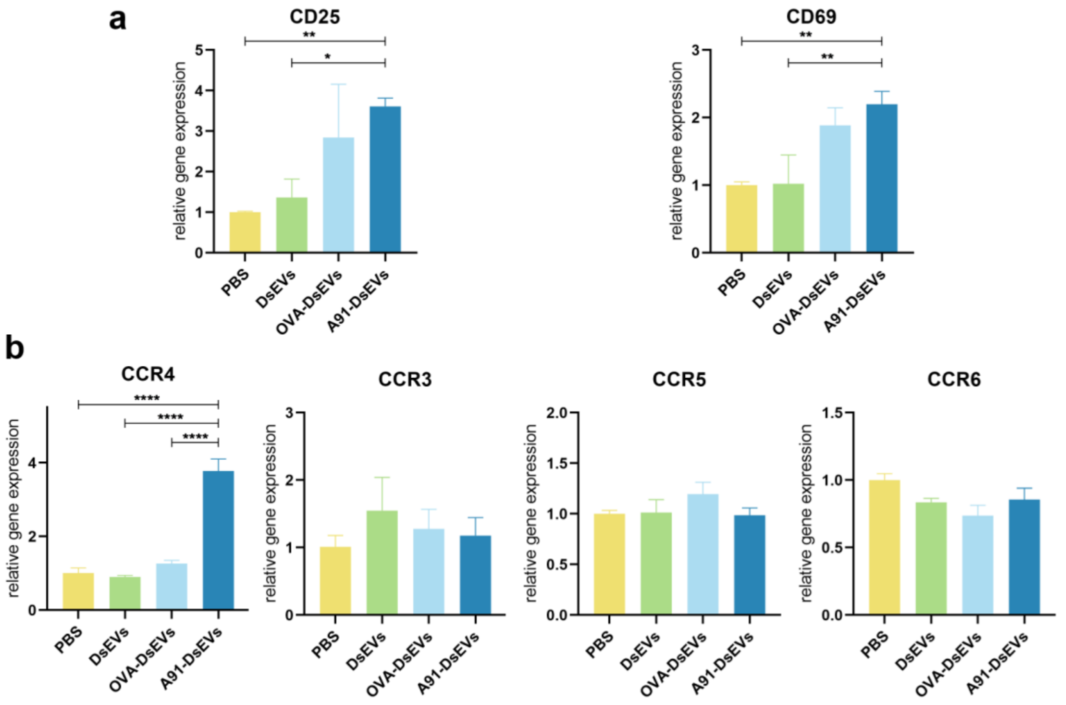

Figure S6. **A91-DsEVs increased the gene expression level of activation markers and migratory chemokine receptors in splenic CD4$^+$ T cells.** The qRT-PCR results showed an upregulation in gene expression of CD25, CD69 and CCR4 in A91-DsEVs stimulated CD4$^+$ T cells (n=3). Data were expressed as mean ± SD. (* $p<0.05$, ** $p<0.01$, *** $p<0.001$, **** $p<0.0001$ as assessed by One-way ANOVA with Dunnett's multiple comparisons).

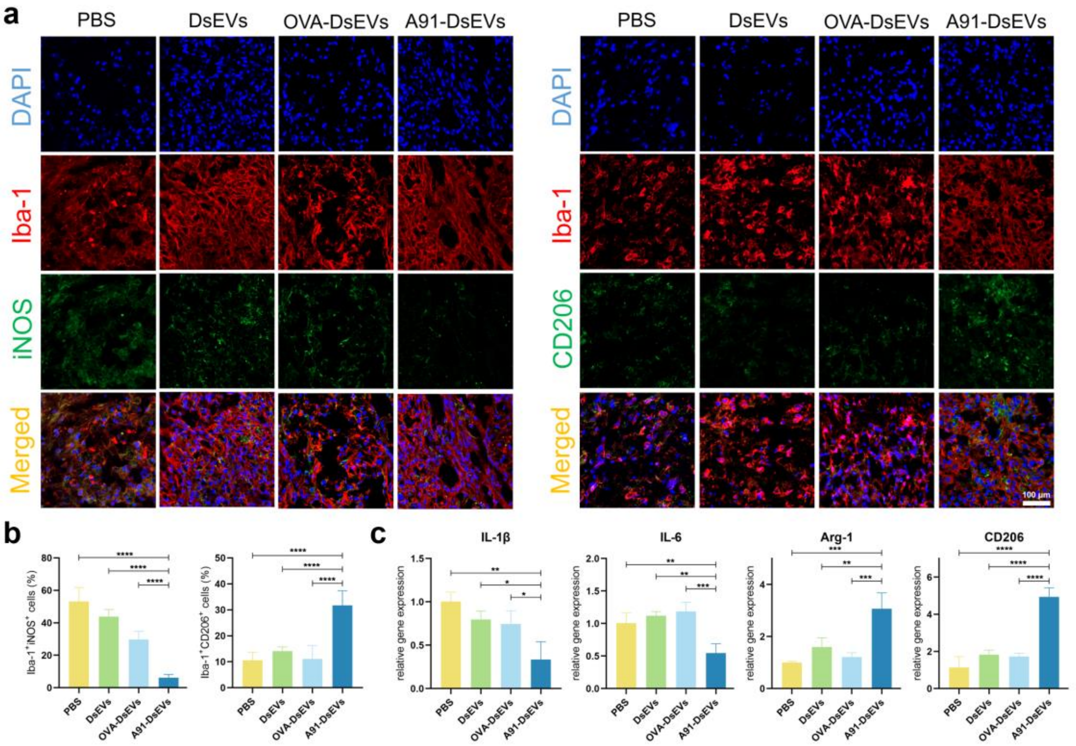

Figure S7. A91-DsEVs led to the polarization of microglia in lesion site. (a) Immunofluorescence images showed the distribution of different subtypes of microglia in the injury site at 7 dpi. (b) The bar chart showed the ratio of $iNOS^{+}Iba\text{-}1^{+}$ cells and $CD206^{+}Iba\text{-}1^{+}$ cells to $Iba\text{-}1^{+}$ cells in different groups. (Scale Bar=100 μm, n=5). (c) The qRT-PCR results showed the gene expression of M1-type markers and M2-type markers in the injury site at 7 dpi (n=3). Data were expressed as mean ± SD. (* $p<0.05$, ** $p<0.01$, *** $p<0.001$, **** $p<0.0001$ as assessed by One-way ANOVA with Dunnett's multiple comparisons).

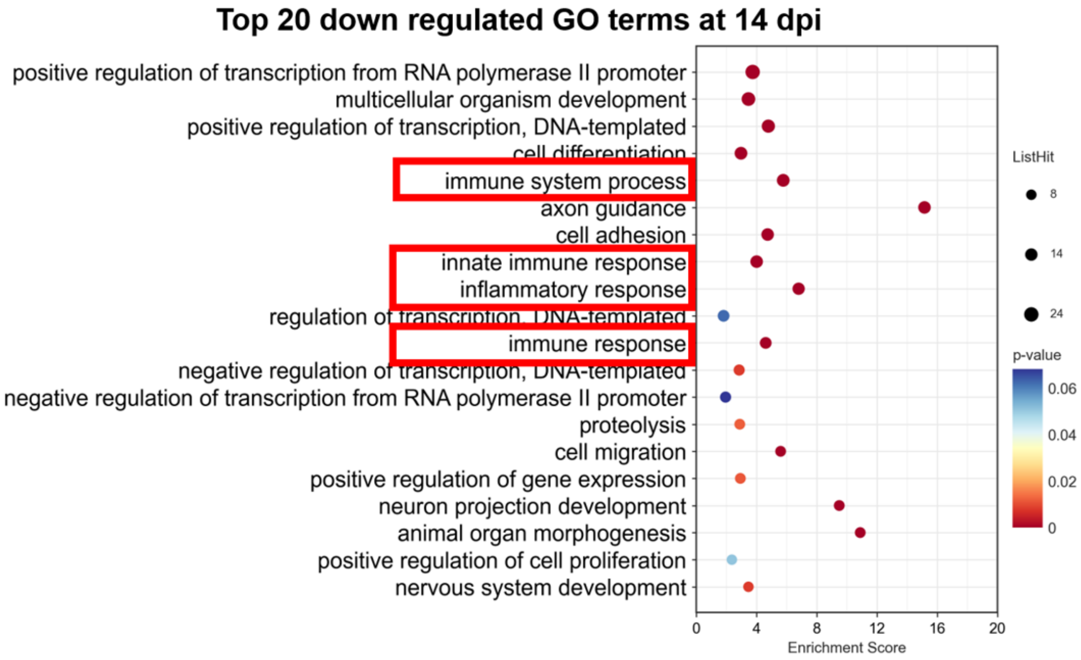

Figure S8. GO enrichment analysis highlighted the downregulated GO terms associated with immune and inflammatory responses in the A91-DsEVs-treated group compared to the PBS-treated group at 14 dpi. Data were expressed as mean ± SD. (* $p<0.05$, ** $p<0.01$, *** $p<0.001$, **** $p<0.0001$ as assessed by two-tailed t-test).

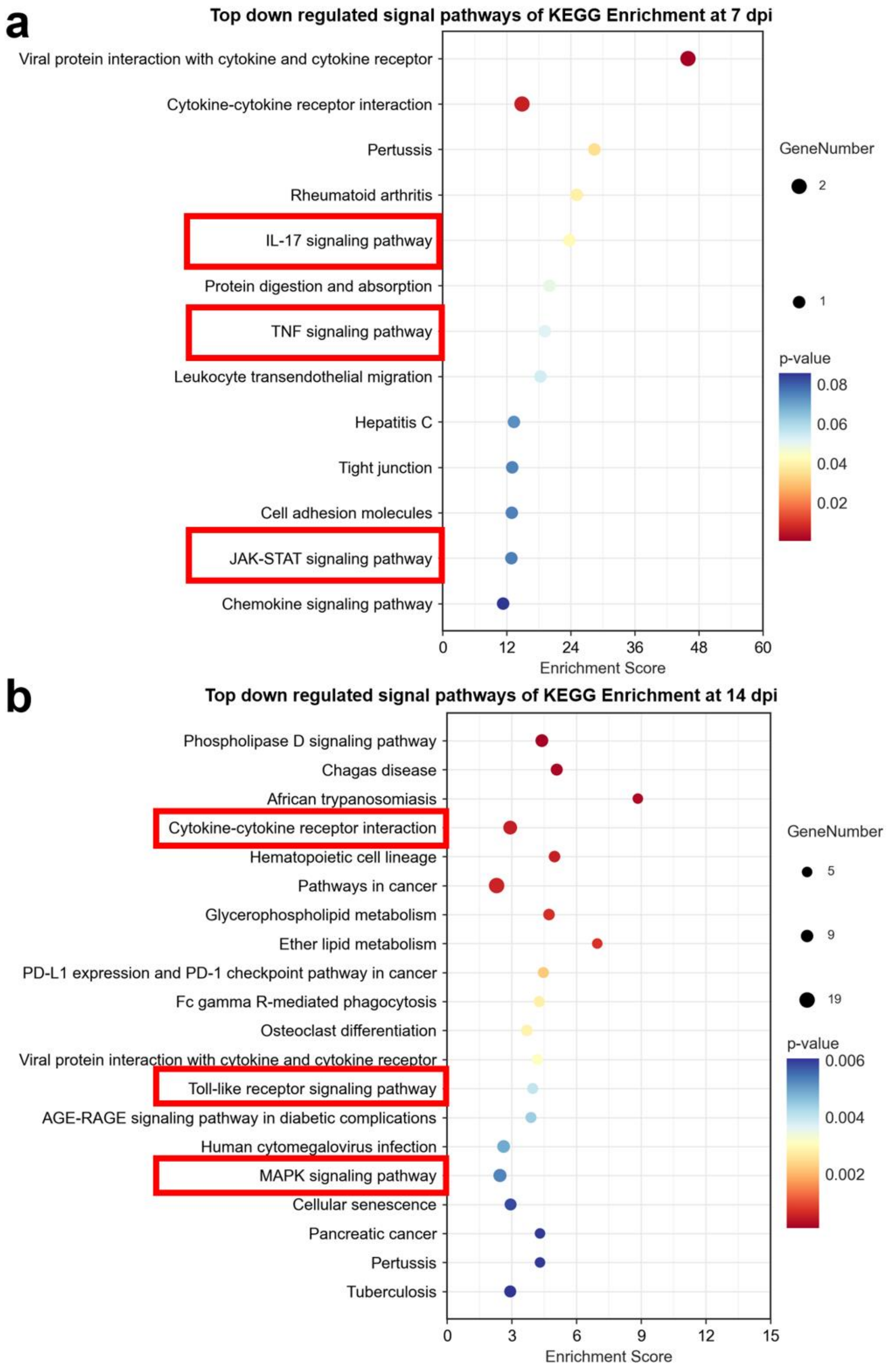

Figure S9. KEGG pathway enrichment analysis of spinal cord in the A91-DsEVs-treated group and the PBS-treated group at 7 and 14 dpi. (a) KEGG pathway enrichment analysis illustrated the downregulated signal pathways in the A91-DsEVs-treated group compared to the PBS-treated group at 7 dpi. (b) KEGG pathway enrichment analysis illustrated the downregulated signal pathways in the A91-DsEVs-treated group compared to the

PBS-treated group at 14 dpi. Data were expressed as mean ± SD. (* $p<0.05$, ** $p<0.01$, *** $p<0.001$, **** $p<0.0001$ as assessed by two-tailed t-test).

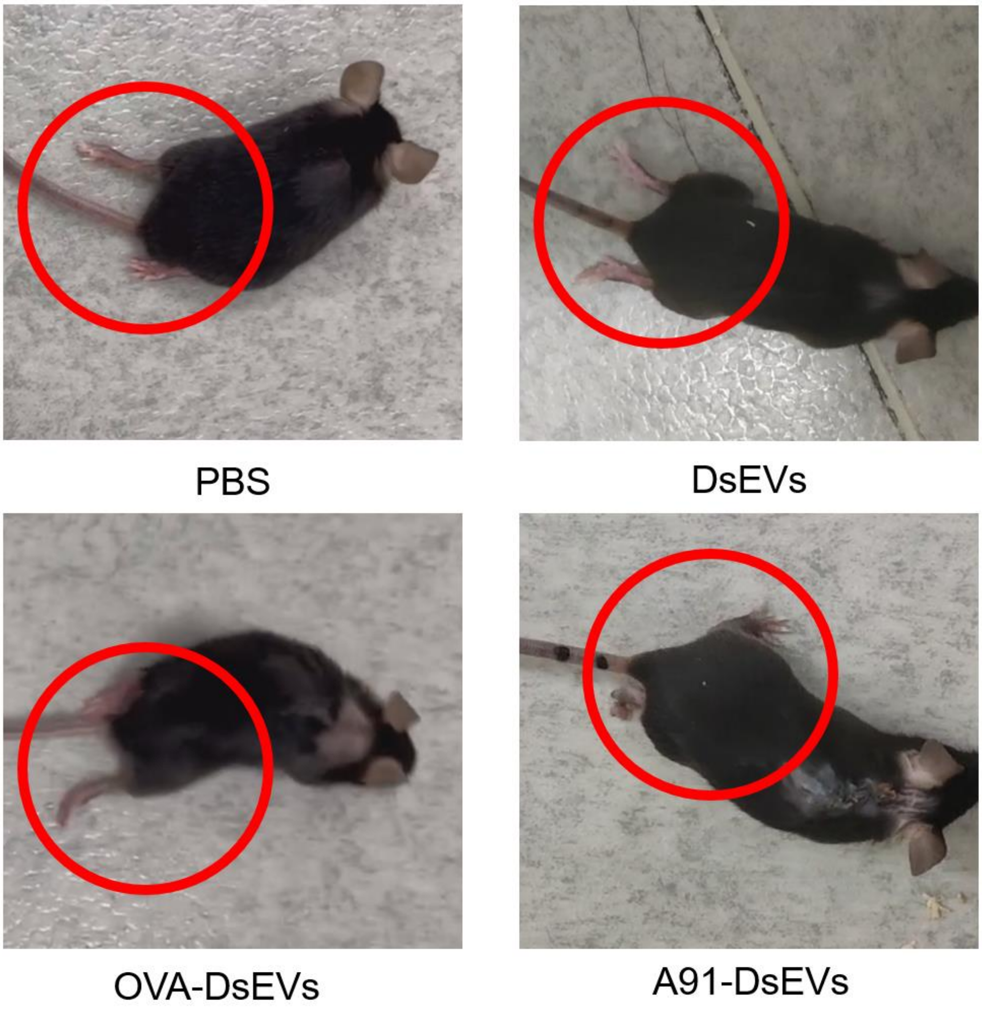

Figure S10. Mice hind limb motor function was improved after A91-DsEVs treatment. The images showed a better recovery of hind limb function was achieved in mice from A91-DsEVs-treated group at 35 dpi.

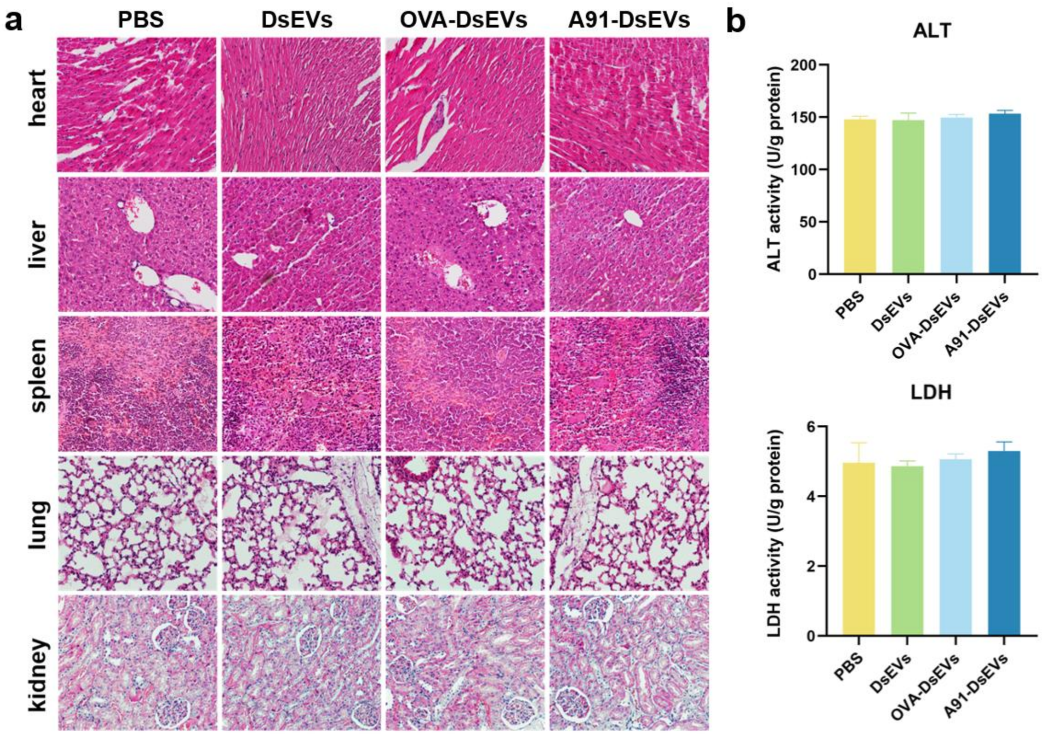

Figure S11. The assessment of the biotoxicity of A91-DsEVs in vivo. (a) HE staining results showed the normal shape of major organs in different treated mice at 3 dpi. (b) The result showed the similar expression levels of LDH and ALT in mice liver from different groups. Data were expressed as mean ± SD. (* $p<0.05$, ** $p<0.01$, *** $p<0.001$, **** $p<0.0001$ as assessed by One-way ANOVA with Dunnett's multiple comparisons).
